# Crellasterones A and B: A-Norsterol Derivatives from the New Caledonian Sponge *Crella incrustans*

**DOI:** 10.3390/md15060177

**Published:** 2017-06-15

**Authors:** Kavita Ragini, Andrew M. Piggott, Peter Karuso

**Affiliations:** Department of Chemistry & Biomolecular Sciences, Macquarie University, Sydney NSW 2109, Australia; kavita.ragini@students.mq.edu.au (K.R.); andrew.piggott@mq.edu.au (A.M.P.)

**Keywords:** steroid, crellasterone, *Crella*, sponge, DFT, marine natural products

## Abstract

Two new steroids, crellasterones A (**1**) and B (**2**), together with the previously reported compound chalinasterol (**3**) and several nucleosides (**4**–**7**), were isolated from the sponge *Crella incrustans*, collected in New Caledonia. The structures of the new compounds were established by extensive NMR and mass spectroscopic analysis and revealed unprecedented marine natural products with a ring-contracted A-norsterone nucleus and 2-hydroxycyclopentenone chromophore. The absolute configurations were derived from electronic circular dichroism (ECD) measurements in conjunction with high-level density functional theory (DFT) calculations.

## 1. Introduction

Sterols [1] are among the most studied groups of natural products, with interest commencing in the 19th century, running through to the 1969 Nobel Prize in Chemistry for Sir Derek Barton, and continuing to the present day. Natural products based on sterols first began to be isolated from terrestrial and marine sources several decades after the initial discovery and characterization of cholesterol [1] and include important compounds such as aplysterol [2] and 25-methylxestosterol [3]. Sponges were the first invertebrates shown to contain sterols other than cholesterol, and since then, sponges have been shown to have the most diverse array of novel sterols [4]. The presence of sterols in sponges is not only interesting from a biochemical and functional viewpoint, but also provides information relevant to the classification of these organisms (chemotaxonomy) [5]. Sponge sterols often contain unusual side-chains and modified ring systems, ranging in carbon content from C_24_ to C_31_ [4,5,6,7].

Sponges from the genus *Crella* have not been widely studied, with only a few natural products isolated, including crellastatins A–M, [8,9,10] shishicrellastatins A and B [11], norselic acids A–E [12] and benzylthiocrellidone [13]. Crellastatin A, a dimeric 4,4′-dimethylsterol from *Crella* sp. exhibits in vitro cytotoxic activity against a human bronchopulmonary non-small-cell lung carcinoma cell line (NSCLC-N6) at an IC_50_ of 1.5 μg/mL [8]. Similarly, crellastatins B–M also exhibit in vitro antitumor activity against NSCLC cell lines with IC_50_ values of 1–10 μg/mL [9,10]. The closely related shishicrellastatin A, and from *Crella spinulata*, B, are cathepsin B inhibitors with an IC_50_ value of 8 μg/mL each [11]. Up regulation of cathepsin B is correlated with tumor cell invasiveness. Norselic acid A [12] is an anti-infective steroid isolated from the Antarctic sponge *Crella* sp. that possesses antibiotic activity against methicillin-resistant *Staphylococcus aureus* (MRSA), methicillin-sensitive *S. aureus* (MSSA), vancomycin-resistant *Enterococcus faecium* (VRE), and *Candida albicans* [12]. Noreselic acids A–E were also found to be active against the *Leishmania* parasite at low micromolar levels. Benzylthiocrellidone, the only non-steroidal metabolite from *Crella* spp., was isolated from *Crella spinulata*, found on the Great Barrier Reef in Australia [13].

**Scheme 1:**
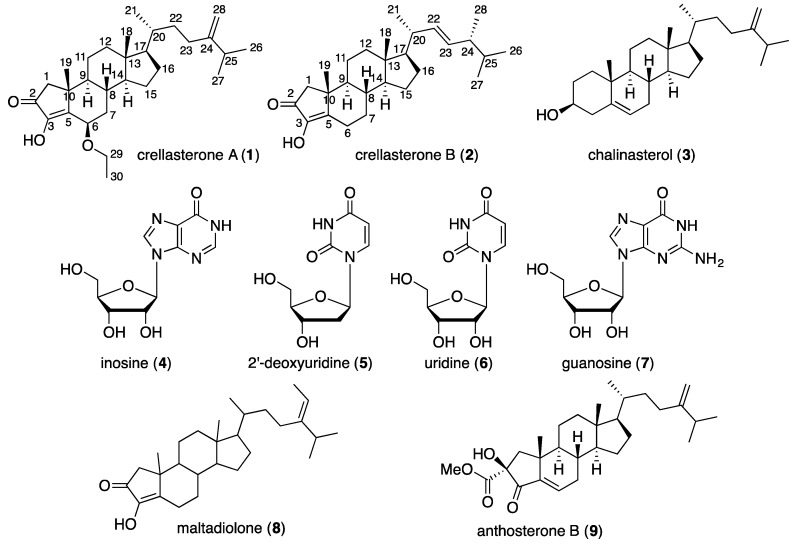
Structures of compounds isolated from *Crella incrustans* (**1**–**7**) in the current study and related compounds (**8**–**9**).

In our search for new marine natural products, two new C_27_ sterols (**1**–**2**) possessing an unusual A-nor skeleton [14] were isolated from the sponge *Crella incrustans* (order Poecilosclerida, family Crellidae) collected from New Caledonia. Herein we describe the isolation and structure elucidation of these novel compounds, along with several previously reported metabolites (**3**–**7**) (Scheme 1).

## 2. Results and Discussion

The frozen sponge was exhaustively extracted with ethanol to afford an extract that was successively partitioned with *n*-hexane/aqueous ethanol, ethyl acetate/aqueous ethanol and *n*-butanol/water. Reversed phase chromatography of the ethyl acetate extract yielded the nucleosides inosine (**4**), 2′-deoxyuridine (**5**), uridine (**6**) and guanosine (**7**). The *n*-hexane extract yielded chalinasterol (**3**) as the major metabolite, along with smaller quantities of two new compounds, crellasterones A (**1**) and B (**2**).

**Figure 1:**
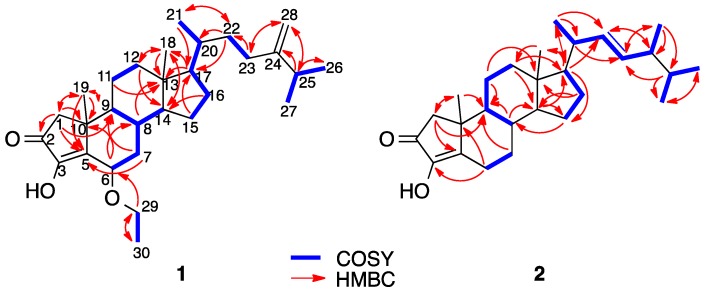
Key HMBC and COSY correlations of crellasterones A (**1**) and B (**2**).

Crellasterone A (**1**) was isolated as an optically active white solid. HRESIMS analysis revealed a protonated molecule [M + H]^+^ at *m*/*z* 443.3524, corresponding to a molecular formula C_29_H_46_O_3_ (∆mmu 0.4). The ^1^H and ^13^C NMR spectra (Table 1) showed resonances for five non-protonated carbons, including one ketone, nine methines, nine methylenes (including one exomethylene), and six methyl groups. Analyses of the 1D and 2D NMR spectra of **1** (COSY, HSQC, HMBC, H2BC) established a sterol-based skeleton. Characterization began with H_2_-1 (*δ*_H_ 2.23, 2.16; *δ*_C_ 48.4), which showed HMBC correlations to the ketone carbonyl C-2 (*δ*_C_ 202.6), as well as the hydroxyenol C-5 (*δ*_C_ 149.5), the methyl C-19 (*δ*_C_ 21.7) and the methine C-9 (*δ*_C_ 53.7). HMBC correlations from H_3_-19 (*δ*_H_ 1.25) to C-1 (*δ*_C_ 48.4), C-5, C-9 and C-10 (*δ*_C_ 40.6) allowed the A-ring to be assigned as 2-hydroxycyclopentenone (Figure 1). IR bands at 3176 (O–H), 1710 (C=O) and 1670 (C=C) cm^−1^ supported this assignment [15], as did the UV spectrum (λ_max_ 265 nm). The remaining steroid rings B–D could be accounted for by HMBC (Figure 1) and H2BC (Supplementary Materials, Table S1) correlations. Ring B was assigned by HMBC correlations from H-6 (*δ*_H_ 4.59) to C-8 (*δ*_C_ 31.3) and C-10 (*δ*_C_ 40.6), and from H_2_-7 (*δ*_H_ 2.03, 1.14) to C-5 (*δ*_C_ 149.5) and C-9 (*δ*_C_ 53.7). H2BC correlations were also observed from H-6 to C-7 (*δ*_C_ 38.0) and from H_2_-7 to C-6 (*δ*_C_ 69.4) and C-8. Methylene H_2_-29 (*δ*_H_ 3.41, q) showed a COSY correlation to methyl H_3_-30 (*δ*_H_ 1.16, t) and HMBC correlations to C-6 and C-30 (*δ*_C_ 15.2), suggesting the presence of an ethoxy group attached to C-6 in ring B. This was supported by a ROESY correlation between H-6 and H_2_-29. Ring C was assigned by HMBC correlations from H_2_-11 (*δ*_H_ 1.56, 1.16) to C-13 (*δ*_C_ 43.1) and H2BC correlations to C-9 (*δ*_C_ 53.7) and C-12 (*δ*_C_ 39.6). Methylene H_2_-12 (*δ*_H_ 1.99, 1.14) showed HMBC correlations to C-14 (*δ*_C_ 55.9) and C-18 (*δ*_C_ 12.2), while methine H-14 (*δ*_H_ 1.11) showed an HMBC correlation to methyl C-18. Furthermore, H_3_-18 (*δ*_H_ 0.72) showed HMBC correlations to C-12, C-13 and C-14. HMBC correlations from H_2_-15 (*δ*_H_ 1.58, 1.34) to C-17 (*δ*_C_ 55.9), from H_2_-16 (*δ*_H_ 1.83, 1.26) to C-14 and from H-17 (*δ*_H_ 0.96) to C-13 and C-18 (*δ*_C_ 12.2) confirmed the assignment of ring D. The side chain of **1** was determined from analysis of the remaining proton and carbon signals. The olefinic protons from the exomethylene H_2_-28 (*δ*_H_ 4.69, 4.63) displayed HMBC correlations to C-23 (*δ*_C_ 31.0) and C-25 *δ*_C_ 33.8). Methine H-25 (*δ*_H_ 2.20) in turn showed HMBC correlations to C-24 (*δ*_C_ 156.8) and C-28 (*δ*_C_ 106.0). The methyl protons H_3_-26 (*δ*_H_ 1.00) showed HMBC correlations to C-24, C-25 and C-27 (*δ*_C_ 21.9). The placement of the double bond at C-24 was based on these key HMBC correlations. Finally, the HMBC correlations from H_2_-23 (*δ*_H_ 2.07, 1.86) to C-28 (*δ*_C_ 106.0) and C-22 (*δ*_C_ 34.6), which could be followed back to the steroidal nucleus by HMBC correlations from H_2_-22 (*δ*_H_ 1.51, 1.12) to C-17 (*δ*_C_ 55.9), C-20 (*δ*_C_ 35.7) and C-21 (*δ*_C_ 18.7), confirmed the side chain assignments.

Crellasterone B (**2**) was also isolated as an optically active white solid. HRESIMS analysis of **2** revealed a protonated molecule [M + H]^+^ at *m*/*z* 399.3263, indicative of a molecular formula C_27_H_42_O_2_ (∆mmu 0.5). IR absorption bands at 3274, 1700 cm^−1^ suggested the presence of hydroxy and carbonyl functionalities. Analysis of the 1D and 2D NMR spectra of **2** (COSY, HSQC, HMBC) revealed the presence of the same steroidal ring system as in **1** (Figure 1). The ^13^C NMR spectrum of **2** displayed fewer carbon resonances relative to **1**, and especially notable was the absence of an oxygen-bearing methine and the ethyl group of **1**, which indicated the absence of the ethoxy group. The ^13^C NMR spectrum also revealed six non-protonated carbons, nine methines, seven methylenes and six methyl carbons. The other notable difference from **1** was in the side chain, with the absence of an exomethylene and the presence of two mutually coupled olefinic methines H-22 (*δ*_H_ 5.13) and H-23 (*δ*_H_ 5.14). Key HMBC correlations from H_3_-21 (*δ*_H_ 0.97) to C-17 (*δ*_C_ 55.8), C-20 (*δ*_C_ 40.3) and C-22 (*δ*_C_ 135.9), and from H_3_-28 (*δ*_H_ 0.89) to C-23 (*δ*_C_ 132.0), C-24 (*δ*_C_ 43.1) and C-25 (*δ*_C_ 33.2) confirmed the positioning of the double bond, between C-22 and C-23. The *dd* coupling of H-23 further implied a methine group at C-24 and confirmed that two methyl groups were attached to C-25. The geometry of the double bond was determined to be *E* (^3^*J*_22,23_ = 15.2 Hz). As with **1**, C-20 was connected back to the steroid nucleus via HMBC correlations (Figure 1 and Supplementary Materials, Table S2), resulting in crellasterone B being assigned structure **2**.

**Figure 2:**
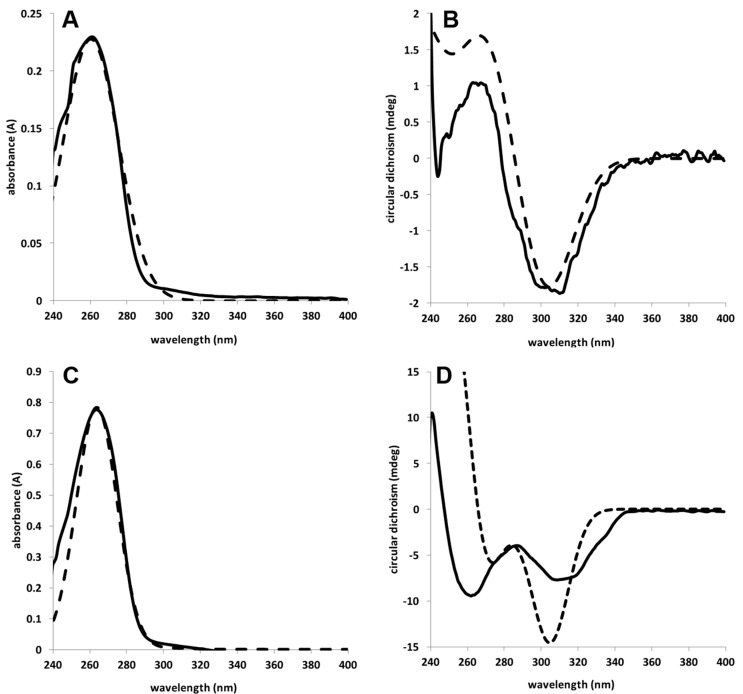
UV-Vis and electronic circular dichroism (ECD) spectra of crellasterones A (**1**) and B (**2**). (**A**) UV-Vis spectrum of **2** (solid line) and calculated spectrum (dashed line) red shifted by 4 nm; (**B**) ECD spectrum of **2** (solid line) and calculated ECD (dashed line); (**C**) UV-Vis spectrum of **1** (solid line) and calculated spectrum (dashed line); (**D**) ECD spectrum of **1** (solid line) and calculated ECD spectra (dashed lines). All calculations were performed using Turbomole 7.1 [16], DFT-D3//PBE0/TZVPP-COSMO (CHCl_3_)—see Supplementary Materials.

Having established the structures of crellasterone A and B, we turned our attention to determination of their absolute stereochemistries using electronic circular dichroism (ECD). The simpler compound **2**, showed a small negative specific rotation and a positive Cotton effect at 263 nm, and a negative Cotton effect at 310 nm in the ECD spectrum (Figure 2B). Since there are no ECD spectral data reported for any related compounds, the ECD spectra could not be compared to literature values. Time-dependent density functional theory (TD-DFT) calculations (Turbomole 7.1) [16] of **2** gave very good matches for UV-Vis (Figure 2A) and ECD (Figure 2B) spectra for (10*R*)-crellasterone B (see Supplementary Materials, Figure S18). The negative Cotton effect at 300 nm and positive Cotton effect at 260 nm were confirmed to arise solely from the A-ring chromophore and the configuration at C-10 by repeating the calculation with just rings A and B (data not shown), which gave identical UV and ECD spectra.

**Figure 3:**
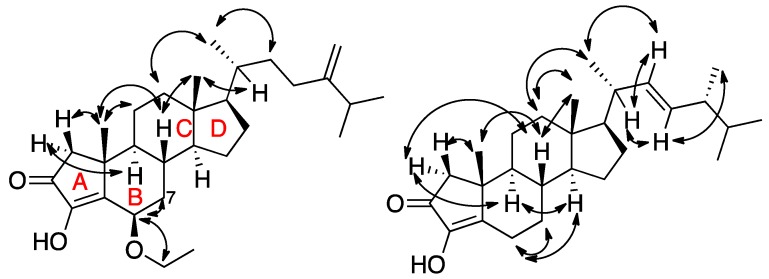
Key ROESY correlations for crellasterones A (**1**) and B (**2**).

Compound **1** is more complex, having a second stereocenter adjacent to the chromophore. While NMR spectroscopy had established the configuration at C-6, we wished to confirm this through TD-DFT calculations. Compound **1** also showed a negative specific rotation and a similar UV-Vis spectrum (Figure 2C), but unlike **2**, negative Cotton effects at 264 and 310 nm in the ECD spectrum (Figure 2D). From the NMR spectrum, H-6 showed small couplings to H-7α and H-7β, suggesting H-6 was equatorial; thus a β-ethoxy group was placed at C-6. TD-DFT calculations of 6α- and 6β-isomers of **1** (Supplementary Materials, Figures S19–S21), supported the assignment of H-6 as α. Only the 6β-ethoxy isomer of **1** gave negative Cotton effects at 301 and 262 nm, in agreement with the experimental ECD spectrum of **1** (Figure 2D and Figure S21). Having established the absolute configurations at C-6 and C-10, ROESY data established the relative configurations of the remaining steroidal core of **1** (Figure 3) [17]. Correlations between H_3_-19, H-11β and H-8 demonstrated the *trans*-relationship of the B/C ring junction and placed the C-19 methyl group on the same face of the steroid ring as H-8. Other significant correlations observed were between H-1β and H-19, and between H-1α, H-7α and H-9, confirming that H-9 is on the α-face. The C/D ring junction was assigned based on ROESY correlations between H-8 and H-18, confirming H-18 and H-8 to be on the β-face. Due to overlapping of peaks, no correlation between H-14 and H-9 could be observed. A ROESY correlation between H-20 and H-18 suggested C-20 was *R* in accord with standard steroidal stereochemistry (Supplementary Materials, Figure S22). The ROESY correlations described above are consistent with the *trans/trans* B/C/D relationship of a regular steroid [12].

Similar to **1**, ROESY data (Figure 3) established configurational assignments of the steroidal core of **2**. Correlations between H_3_-19 and H-8, and between H-8 and H-18, established the *trans/trans* relationship of the B/C/D ring, placing H-8 and C-18 methyl group on the same face of the steroid ring as C-19 methyl group. Other significant ROESY correlations observed were between H-1β and H_3_-19 (above the plane of the ring system), between H-1α, H-9 and H-11α, and between H-12α and H-21 (below the plane). The C-24 configuration of **2** was assigned as *S* due to the fact that the chemical shift difference between C-26 and C-27 carbon atoms was 0.5 ppm and the chemical shift of C-28 was 18.1 ppm, in accordance with the rules developed by Wright [18].

Compounds **1** and **2** are C_27_ sterols related to maltadiolone (**8**) and its esters, an unpublished series of semi-synthetic steroids that appear in a patent without any stereochemistry or spectral data [19]. These compounds belong to the small group of A-nor ring-contracted steroids [20,21]. The first example of a ring A-contracted steroid, bearing a one-carbon appendage at C-2 (anthosterones A and B (**9**)), was described by Andersen, Clardy and co-workers in 1988 as a marine natural product from *Anthoracuata (Antho) graceae* [22] and the same compounds, plus a range of side chain analogues, were isolated by Molinki in 2004 from *Phorbas amaranthus* and displayed moderate cytotoxicity against HCT-116 tumor cells [21]. Both sponges are in the same order as *Crella* (Poecilosclerida) and the latter is synonymous with *Crella hospitalis* (Schmidt, 1870) [23]. A compound with an A-nor-cholesterol core identical to that of crellasterone B, namely 3-hydroxy-4-nor-5α-cholest-3-en-2-one, but with a different side chain was reported as a side-product of bis-steroid synthesis [24]. However, the reported structure is certainly incorrect as the reported ^13^C chemical shifts of C-3 and C-5 (*δ*_C_ 184.8 and 125.9 respectively) are not reasonable for 2-hydroxycyclopent-2-enone and would fit better for cyclopent-2-enone.

**Figure 4:**
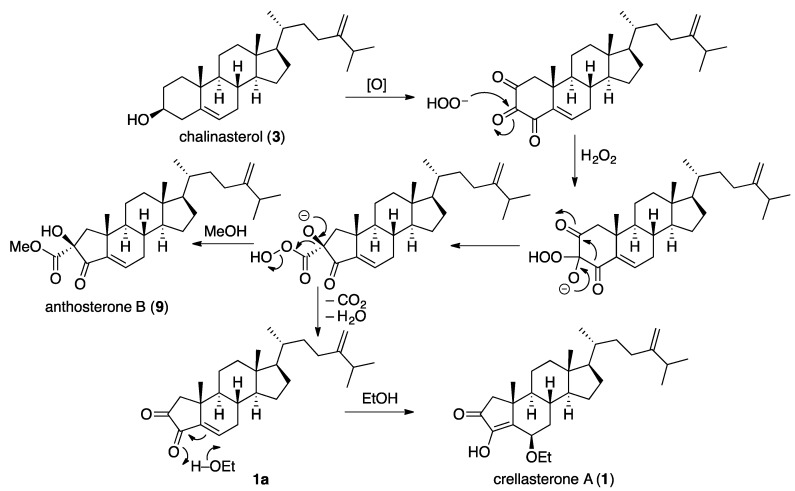
Proposed biogenesis of crellasterone A (**1**) from chalinasterol (**3**) that also accounts for the presence of anthosterones in related sponges.

It is likely that **1** is the reaction product of an electrophilic natural product, such as **1a**, with ethanol, the extraction solvent (Figure 4). A similar proposal has been shown by us in relation to the abiotic synthesis of an ethoxy substituted spiroisoxazoline alkaloid and furanones [25,26]. Similarly, the anthosterones (e.g., **9**) could arise from the abiotic reaction of an intermediate peracid with methanol (Figure 4). A possible biogenesis of **1** and **2** from chalinasterol (3β-ergosta-5,24(28)-dien-3-ol) is via a Baeyer–Villiger-type oxidative ring contraction and decarboxylation (Figure 4) [27]. Using the same mechanism, but starting with spongosterol ((24*S*)-5α-ergost-22*E*-en-3β-ol) instead of chalinasterol, would lead to crellasterone B (**2**).

In this sponge, the known compound chalinasterol (**3**) was present as the major component, making up 5% of the *n*-hexane extract. The structure was identified by comparison with published spectroscopic data [28]. The polar fractions contained nucleosides, including inosine (**4**), 2′-deoxyuridine (**5**), uridine (**6**) and guanosine (**7**), (Scheme 1) identified on the basis of ^1^H NMR spectra and mass spectrometry.

## 3. Experimental Section

### 3.1. General Experimental Procedures

Optical rotations were measured on a Jasco P-1010 Polarimeter (Jasco, Tokyo, Japan) and electronic circular dichroism spectra were acquired on a Jasco J-810 spectropolarimeter (Jasco). Infrared spectra were recorded on a Nicolet iS10 ATR FTIR Spectrometer (Thermo Scientific, Waltham, MA, USA). NMR spectra were acquired at 25 °C on a Bruker Avance AVII 600 MHz NMR spectrometer in CDCl_3_, processed using Bruker Topspin 3.5 software (Bruker, Fällanden, Switzerland) and referenced to residual solvent (CHCl_3_ *δ*_H_ 7.24; *δ*_C_ 77.01). LC–MS was performed on an Agilent 1260 (Agilent, Santa Clara, CA, USA) Infinity UHPLC coupled to an Agilent 6130 single quadrupole mass spectrometer by electrospray ionization in positive and negative polarity modes. UV-Vis spectra were acquired on an Agilent 1260 Infinity diode array detector. High-resolution mass spectra were acquired on a Thermo Scientific LTQ Orbitrap XL or a Q Exactive Plus hybrid quadrupole-Orbitrap mass spectrometer (ThermoFisher Scientific, Waltham, MA, USA) by direct infusion in a positive polarity mode. HPLC separations were achieved on a Gilson 506C HPLC system (Gilson, Middleton, WI, USA) with UNIPOINT v5.11 software. Analytical and preparative HPLC were performed on Synergi Max-RP HPLC columns (Phenomenex): Synergi 10 µ MAX-RP, 250 × 4.6 mm and Synergi 10 µ MAX-RP, 250 × 21.2 mm respectively. LC–MS was performed on a C_18_ analytical column (Phenomenex Gemini 3 µ C18, 150 × 2.0 mm) with a gradient from 5 to 100% CH_3_CN in H_2_O with 0.025% formic acid; flow rate 0.2 mL/min and UV detection at 254 nm.

### 3.2. Animal Material

The sponge was collected by self-contained underwater breathing apparatus (SCUBA) in New Caledonia. The samples were frozen upon collection, and stored at −20 °C. The sponge was identified as *Crella incrustans* (class Demospongiae, order Poecilosclerida, family Crellidae). A voucher specimen under the registration number (NC-10) is kept in the collections at the University of Auckland, New Zealand. Another voucher specimen has been deposited at the Department of Chemistry & Biomolecular Sciences, Macquarie University, Sydney, Australia, also under NC-10.

### 3.3. Extraction and Isolation

The frozen sponge (840 g) was homogenized in EtOH (3 × 1 L) and the combined EtOH extracts were filtered and evaporated to approximately 1 L. The extract was partitioned with *n*-hexane (3 × 500 mL), ethyl acetate (3 × 500 mL), and *n*-butanol (2 × 200 mL) after removal of the remaining ethanol to yield 2.6 g, 2.6 g and 1.4 g of crude extracts respectively. The *n*-hexane extract was subjected to gel permeation chromatography (Biobeads SX-3; 480 × 22 mm with toluene), to afford four fractions (H1–H4). Fractions H2 and H3 were combined and fractionated on a silica gel flash column with a stepwise gradient from 100% *n*-hexane to 100% EtOAc to afford seven subfractions (F1–F7). From the oily fraction F2 (353.6 mg), 6.5 mg of **1** precipitated out of methanol. F3 (111.1 mg) was purified by reversed phase HPLC (Synergi RP-Max C12, 10 µm, 250 × 22 mm) using a gradient from 75 to 100% CH_3_CN in H_2_O 40 min to afford compound **2** (*t*_R_ 35.5 min; 2.5 mg). Compound **3** (112.2 mg) crystalized from fractions F4 and F5. The ethyl acetate extract was subjected to gel permeation chromatography (Sephadex LH-20; 1:1 chloroform:methanol), to afford four main subfractions (E1–E4). Fractions E3 and E4 were individually passed through an Alltech high capacity C_18_ solid-phase extraction cartridge, eluting with 90% CH_3_CN in H_2_O, and the eluates were purified separately by preparative HPLC column (Synergi RP-Max C12, 10 µm, 250 × 22 mm) using an isocratic 5% MeOH in H_2_O over 30 min and UV detection at 300 nm. Fraction E3 (321.3 mg) afforded compounds **4** (*t*_R_ 16.8 min; 2.7 mg) and **5** (*t*_R_ 12.4 min; 2.8 mg), and fraction E4 (82.5 mg) afforded compounds **6** (*t*_R_ 10.5 min; 5.6 mg) and **7** (*t*_R_ 20.0 min, 2.7 mg). The *n*-butanol extract contained mostly salt and was not investigated further.

*Crellasterone A [3-hydroxy-A-norergo-3(5),22-dien-2-one]* (**1**): white solid (6.5 mg); ${\left[\alpha \right]}_{D}^{20}$ −33 (*c* 0.65, CHCl_3_); UV (CHCl_3_) *λ*_max_ (log ε) 264 (4.31) nm; ECD (*c* 0.04 mM, CHCl_3_) *λ*_max_ (Δε) 264 (−7.57), 310 (−6.21) nm; IR (neat film) ν_max_ 3176, 2950, 2962, 1710, 1670, 1404 cm^−1^; ^1^H and ^13^C NMR data, see Table 1; ESIMS *m*/*z* 443 [M + H]^+^; HRESIMS *m*/*z* 443.3524 [M + H]^+^ (calculated for C_29_H_47_O_3_^+^, 443.3520).

*Crellasterone B [3-hydroxy,6β-ethoxy-A-norergo-3(5),24(28)-dien-2-one]* (**2**): white solid (2.9 mg); ${\left[\alpha \right]}_{D}^{20}$ −4 (*c* 0.29, CHCl_3_); UV (CHCl_3_) *λ*_max_ (log ε) 261 (2.84) nm; ECD (*c* 0.32 mM, CHCl_3_) *λ*_max_ (Δε) 263 (+0.08), 310 (−0.20) nm; IR (neat film) ν_max_ 3274, 2954, 2923, 1700, 1661, 1460 cm^−1^; ^1^H and ^13^C NMR data, see Table 1; ESIMS *m*/*z* 399 [M + H]^+^; HRESIMS *m*/*z* 399.3263 [M + H]^+^ (calculated for C_27_H_43_O_2_^+^, 399.3258), *m*/*z* 421.3075 [M + Na]^+^ (calculated for C_27_H_42_NaO_2_^+^, 421.3077).

## 4. Conclusions

Marine sponges of the genus *Crella* have been the subject of relatively few natural products studies, with only six papers published in the past 50 years. Herein, we have reported two new unusual sterols, crellasterones A and B, from *Crella incrustans*, collected in New Caledonia. These sterols represent a new type of A-norsteroid not previously reported as natural products. The absolute configurations of the new compounds were determined on the basis of ECD in conjunction with DFT calculations.

## Figures and Tables

**Table 1:** NMR data (CDCl_3_, ^1^H 600 MHz, ^13^C 150 MHz) for crellasterones A (**1**) and B (**2**).

Position ^a^	Crellasterone A (1)		Crellasterone B (2)	
	*δ*_C_, Type	*δ*_H_ (*J* in Hz)	*δ*_C_, Type	*δ*_H_ (*J* in Hz)
1	48.4, CH_2_	α 2.23, d (18.8)	47.3, CH_2_	α 2.21, d (18.8)
		β 2.16, d (18.8)		β 2.15, d (18.8)
2	202.6, C	-	201.6, C	-
3	147.1, C	-	144.1, C	-
5	149.5, C	-	154.8, C	-
6	69.4, CH	4.59, m	22.4, CH_2_	α 2.81, m
				β 2.12, m
7	38.0, CH_2_	α 2.03, m	31.7, CH_2_	α 1.87 m
		β 1.14, m		β 0.95, m
8	31.3, CH	1.84, m	35.9, CH	1.48, m
9	53.7, CH	0.85, m	53.9, CH	0.86, m
10	40.6, C		40.6, C	
11	23.6, CH_2_	α 1.16, m	23.8, CH_2_	α 1.37, m
		β 1.56, m		β 1.52, m
12	39.6, CH_2_	α 1.99, m	39.5, CH_2_	α 1.97, m
		β 1.14, m		β 1.16, m
13	43.1, C		42.9, C	
14	55.9, CH	1.11, m	55.9, CH	1.12, m
15	24.1, CH_2_	α 1.34, m	24.3, CH_2_	α 1.55, m
		β 1.58, m		β 1.07, m
16	28.2, CH_2_	α 1.83, m	28.8, CH_2_	α 1.67, m
		β 1.26, m		β 1.20, m
17	55.9, CH	0.96, m	55.8, CH	0.99, m
18	12.2, CH_3_	0.72, s	12.3, CH_3_	0.71, s
19	21.7, CH_3_	1.25, s	20.3, CH_3_	1.13, s
20	35.7, CH	1.39, m	40.3, CH	1.99, m
21	18.7, CH_3_	0.92, d (6.6)	21.0, CH_3_	0.97, d (6.6)
22	34.6, CH_2_	α 1.12, m	135.9, CH	5.13, dd (15.2, 7.5)
		β 1.51, m		
23	31.0, CH_2_	α 2.07, m	132.0, CH	5.14, dd (15.2, 7.8)
		β 1.86, m		
24	156.8, C	-	43.1, CH	1.81, m
25	33.8, CH	2.20, m	33.2, CH	1.43, m
26	22.0, CH_3_	1.00, d (3.7)	19.7, CH_3_	0.80, d (6.8)
27	21.9, CH_3_	0.99, d (3.7)	20.2, CH_3_	0.82, d (6.8)
28	106.0, CH_2_	α 4.69, m	18.1, CH_3_	0.89, d (6.8)
		β 4.63, m		
29	64.1, CH_2_	3.41, q (7.0)	-	-
30	15.2, CH_3_	1.16, t (7.0)	-	-

^a^ see Figure 1 for locant numbering.

## Supplementary Materials

Supplementary Materials including NMR spectra, tabulated 2D NMR data, HRMS, 1D and 2D NMR spectra, DFT results are available on-line www.mdpi.com/1660-3397/15/6/177/s1.
