# Supplementary material for: Crellasterones A and B: A-Norsterol Derivatives from the New Caledonian Sponge Crella incrustans
Source: Mar Drugs. 2017 Jun 15;15(6):177. doi: 10.3390/md15060177 (PMC5484127; doi:10.3390/md15060177)
Supplement: Supplementary file 1 [file marinedrugs-15-00177-s001.pdf]

SUPPORTING INFORMATION

Crellasterones A and B: A-norsterol derivatives from the New Caledonian sponge *Crella incrustans*

*Kavita Ragini, Andrew M. Piggott and Peter Karuso**

Department of Chemistry & Biomolecular Sciences, Macquarie University, NSW 2109, Australia

Table of Contents

Characterization of Compounds 2

Table S1. NMR data (600 MHz) for crellasterone A (**1**) in $CDCl_3$.. 3
Table S2. NMR data (600 MHz) for crellasterone B (**2**) in $CDCl_3$.. 4

Figure S1. High resolution mass spectrum of crellasterone A (**1**) 5
Figure S2. High resolution mass spectrum of crellasterone B (**2**) 5
Figure S3. ^{1}H NMR spectrum (600 MHz) of crellasterone A (**1**) in $CDCl_3$ 6
Figure S4. ^{13}C NMR spectrum (150 MHz) of crellasterone A (**1**) in $CDCl_3$ 7
Figure S5. ^{1}H-^{13}C HSQC spectrum (600 MHz) of crellasterone A (**1**) in $CDCl_3$.................... 8
Figure S6. ^{1}H-^{13}C HMBC spectrum (600 MHz) of crellasterone A (**1**) in $CDCl_3$................... 9
Figure S7. ^{1}H-^{13}C H2BC spectrum (600 MHz) of crellasterone A (**1**) in $CDCl_3$ 10
Figure S8. ^{1}H-^{1}H COSY spectrum (600 MHz) of crellasterone A (**1**) in $CDCl_3$.................. 11
Figure S9. ^{1}H-^{1}H ROESY spectrum (600 MHz) of crellasterone A (**1**) in $CDCl_3$................. 12
Figure S10. ^{1}H NMR spectrum (600 MHz) of crellasterone B (**2**) in $CDCl_3$ 13
Figure S11. ^{13}C NMR spectrum (150 MHz) of crellasterone B (**2**) in $CDCl_3$ 14
Figure S12. ^{1}H-^{13}C HSQC spectrum (600 MHz) of crellasterone B (**2**) in $CDCl_3$ 15
Figure S13. ^{1}H-^{13}C HMBC spectrum (600 MHz) of crellasterone B (**2**) in $CDCl_3$............... 16
Figure S14. ^{1}H-^{1}H COSY spectrum (600 MHz) of crellasterone B (**2**) in $CDCl_3$ 17
Figure S15. ^{1}H-^{1}H ROESY spectrum (600 MHz) of crellasterone B (**2**) in $CDCl_3$............... 18
Figure S16. ^{1}H NMR spectrum (600 MHz) of chalinasterol (**3**) in $CDCl_3$............................ 19
Figure S17. ^{13}C NMR spectrum (150 MHz) of chalinasterol (**3**) in $CDCl_3$........................... 20
Figure S18.Coordinate files for crellasterone B... 21
Figure S19.Coordinate files for 6α-crellasterone A.. 22
Figure S20.Coordinate files for 6α-crellasterone A.. 23
Figure S21.Coordinate files for 6β-crellasterone A.. 24
Figure S22. Ten lowest energy structures of **1**, superimposed showing the relative distances between protons.. 25
References 25

Characterization of compounds:

Chalinasterol (**3**):[1] white solid (112.2 mg); $[\alpha]^{20}_{D}$ –28 (*c* 0.50, $CHCl_3$) Lit.[2] $[\alpha]^{20}_{D}$ –23 (*c* 0.1, $CHCl_3$); UV (MeOH) λ_{max} 262 nm; IR (neat film) ν_{max} 3335, 2957, 2935, 1465 cm^{-1}; ^1H NMR ($CDCl_3$, 600 MHz) δ 5.33 (m, H-6), 4.49; 4.63 (m, H-25), 3.50 (m, H-3), 2.27; 2.21 (m, H-3), 2.20 (m, H-26), 2.07; 1.86 (m, H-23), 1.98; 1.13 (m, H-12), 1.95; 1.49 (m, H-7), 1.85; 1.08 (m, H-1), 1.83; 1.24 (m, H-16), 1.82; 1.49 (m, H-2), 1.55; 1.24 (m, H-15), 1.52; 1.13 (m, H-22), 1.46 (m, H-11), 1.42 (m, H-8), 1.39, (m, H-20), 1.10 (m, H-17), 1.00 (m, H-27), 1.00 (m, H-28), 0.99 (m, H-18), 0.98 (m, H-14), 0.93 (m, H-21), 0.91 (m, H-9), 0.66 (m, H-19). ^{13}C-NMR ($CDCl_3$, 150 MHz) δ 156.4 (C-24), 140.3 (C-5), 121.3 (C-6), 105.5 (C-25), 71.3 (C-3), 56.3 (C-14), 55.5 (C-17), 49.6 (C-9), 41.8 (C-4), 41.8 (C-13), 39.1 (C-12), 36.8 (C-1), 36.0 (C-10), 35.3 (C-20), 34.4 (C-22), 33.3 (C-26), 31.4 (C-7), 31.2 (C-2), 31.1 (C-8), 30.5 (C-23), 27.8 (C-16), 23.8 (C-15), 21.5 (C-27), 21.4 (C-28), 20.6 (C-11), 18.9 (C-18), 18.2 (C-21), 11.4 (C-19); Mass spectrum (ESIMS) *m/z*: 399 $[M + H]^+$ for $C_{28}H_{47}O^+$.

Inosine (**4**): white solid (2.7 mg); $[\alpha]^{20}_{D}$ –30 (*c* 0.27, MeOH Lit.[3] $[\alpha]^{20}_{D}$ –59 (*c* 1, H_2O); ^1H NMR (DMSO-d_6, 600 MHz) δ 12.39 (s, H-3), 8.34 (s, H-8), 8.06 (d, J = 4.0 Hz, H-2), 5.48 (s, H-4′-OH), 5.85 (d, J = 5.8 Hz, H-1′), 5.20 (s, H-3′-OH), 5.08 (s, H-5′-OH), 4.47 (t, J = 5.3 Hz, H-2′), 4.11 (dd, J = 1.3, 3.5 Hz, H-3′), 3.92 (q, J = 3.8 Hz, H-4′), 3.64; 3.53 (dd, J = 3.8, 7.8 Hz, H-5′). ^{13}C-NMR (DMSO-d_6, 150 MHz) δ 156.5 (C-6), 148.2 (C-4), 145.9 (C-2), 138.7 (C-8), 124.4 (C-5), 87.4 (C-1′), 85.6 (C-4′), 74.1 (C-2′), 70.3 (C-3′), 61.3 (C-5′); Mass spectrum (ESIMS) *m/z*: 269 $[M + H]^+$ for $C_{10}H_{13}N_4O_5^+$.

2'-deoxyuridine (**5**): white solid (2.8 mg); $[\alpha]^{20}_{D}$ +40 (*c* 0.28, MeOH Lit.[4] $[\alpha]^{20}_{D}$ +30 (*c 2*, H_2O); ^1H NMR (DMSO-d_6, 600 MHz) δ 11.29 (s, H-3), 7.84 (d, J = 8.0 Hz, H-6), 6.13 (t, J = 7.3 Hz, H-1′), 5.62 (dd, J = 2.2, 8.0 Hz, H-5), 5.24 (d, J = 4.2 Hz, H-3′-OH), 5.01 (t, J = 5.2 Hz, H-5′-OH), 4.21 (m, H-3′), 3.76 (q, J = 3.6 Hz, H-4′), 3.53 (m, H-5′), 2.07 (m, H-2′). ^{13}C-NMR (DMSO-d_6, 150 MHz) δ 163.1 (C-4), 150.2 (C-2), 140.5 (C-6), 101.7 (C-5), 87.4 (C-4′), 84.1 (C-1′), 70.4 (C-3′), 61.3 (C-5′), 39.0 (C-2′); Mass spectrum (ESIMS) *m/z*: 223 $[M + H]^+$ for $C_9H_{13}N_2O_5^+$.

Uridine (**6**): white solid (5.6 mg); $[\alpha]^{20}_{D}$ +6 (*c* 0.5, MeOH Lit.[5] $[\alpha]^{20}_{D}$ +4 (*c* 0.5, MeOH); ^1H NMR (DMSO-d_6, 600 MHz) δ 11.30 (s, H-3), 7.87 (d, J = 8.1 Hz, H-6), 5.76 (d, J = 5.4 Hz, H-1′), 5.63 (dd, J = 2.2, 8.1 Hz, H-5), 4.01 (t, J = 5.4 Hz, H-2′), 3.94 (t, J = 4.3 Hz, H-3′), 3.82 (q, J = 3.6 Hz, H-4′), 3.60; 3.53 (dd, J = 3.3, 8.7 Hz H-5′). ^{13}C-NMR (DMSO-d_6, 150 MHz) δ 163.1 (C-4), 150.7 (C-2), 140.7 (C-6), 101.7 (C-5), 87.6 (C-1′), 84.8 (C-4′), 73.5 (C-2′), 69.9 (C-3′), 60.8 (C-5′); Mass spectrum (ESIMS) *m/z*: 245 $[M + H]^+$ for $C_9H_{12}N_2O_6^+$.

Guanosine (**7**): white solid (2.7 mg); $[\alpha]^{20}_{D}$ –36 (*c* 0.27, MeOH Lit.[6] $[\alpha]^{20}_{D}$ –48 (*c* 0.1, H_2O); ^1H NMR (DMSO-d_6, 600 MHz) δ 10.62 (s, H-3), 7.92 (s, H-8), 6.44 (br,s, -NH_2), 5.68 (d, J = 5.9 Hz, H-1′), 5.38 (d, J = 6.1 Hz, H-2′-OH), 5.11 (d, J = 4.8 Hz, H-3′-OH), 5.03 (d, J = 5.5 Hz, H-5′-OH), 4.38 (q, J = 6.1 Hz, H-2′), 4.07 (q, J = 4.8 Hz, H-3′), 3.85 (q, J = 3.9 Hz, H-4′), 3.59; 3.50 (m, H-5′).; Mass spectrum (ESIMS) *m/z*: 284 $[M + H]^+$ for $C_{10}H_{13}N_5O_5^+$.

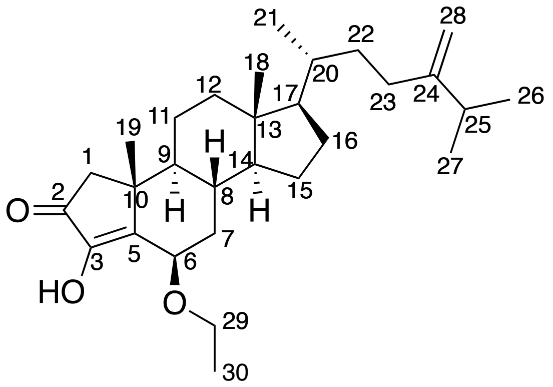

Table S1. NMR data (600 MHz) for crellasterone A (**1**) in $CDCl_3$

Position	δ_C, type	δ_H (*J* in Hz)	COSY	HMBC	H2BC	ROESY
1	47.9, CH_2	α 2.23, d (18.8)	1β	2,3,5,9,10,19		1β,9
		β 2.16, d (18.8)	1α	2,3,5,9,10,19		1α,19
2	202.2, C					
3	149.0, C					
5	146.7, C					
6	68.9, CH	4.59, m	7α,7β	3,5,7,8,10,29	7	7α,7β,29,30
7	37.5, CH_2	α 2.03, m	6,7β,14	3,6,9,14	6,8	6,7β,9
		β 1.14, m	6,7α,8	3,6,9,14	6,8	6,7α,8
8	30.8, CH	1.84, m	7β,9,14	7,9	7,9	7β,11β,18,19
9	53.2, CH	0.85, m	8	12,19	8	1α,7α
10	40.2, C					
11	23.2, CH_2	α 1.16, m	11β,12α	12		11β,12α
		β 1.56, m	11α,12β	9,12,13	9,12	8,11α,12β,18,19
12	39.2, CH_2	α 1.99, m	11α,12β	13,14		11α,21
		β 1.14, m	11β,12α	18		11β
13	42.6, C					
14	55.5, CH	1.11, m	7α,8	12,13,18,20	16	
15	23.7, CH_2	α 1.34, m	15β	8,13,17		15β
		β 1.58, m	15α	8,13,17	14,16	15α,16α,16β
16	27.7, CH_2	α 1.83, m	16β,17	13,14	15,17	15β
		β 1.26, m	16α		15,17	16α,15β
17	55.5, CH	0.96, m	16α,20	8,13,18		21,22α
18	11.8, CH_3	0.72, s		12,13,14		8, 11β,20
19	21.2, CH_3	1.25, s		1,3,9,10		1β,8,11β
20	35.3, CH	1.39, m	17,21	17,21,22	17,21	18
21	18.2, CH_3	0.92, d (6.6)	20	17,20,22	20	12,17,22α
22	34.2, CH_2	α 1.12, m	22β,23α	17	20	17,22β
		β 1.51, m	22α,23β	17,20,21,23	23	22α,23β
23	30.5, CH_2	α 2.07, m	22α,23β	22,24,28	22	23β
		β 1.86, m	22β,23α	22,24,28	22	22β,23α
24	156.4, C					
25	33.3, CH	2.20, m	26,27	24,28	26	26
26	21.5, CH_3	1.00, d (3.7)	25	24,25,27	25	25
27	21.4, CH_3	0.99, d (3.7)	25	24,25,26	25	
28	105.5, CH_2	α 4.69, m	28β	23,24,25		28β
		β 4.63, m	28α	23,24,25		28α
29	63.7, CH_2	3.41, q (7.0)	30	6,30	30	6,30
30	14.7, CH_3	1.16, t (7.0)	29	29	29	6,29

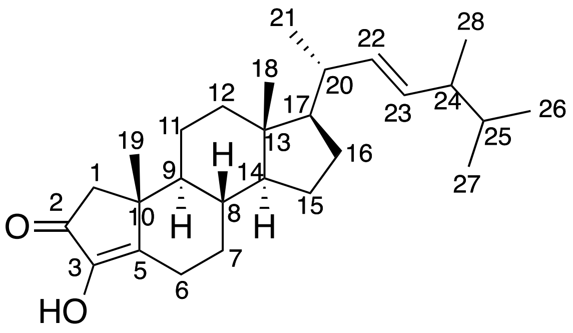

Table S2. NMR data (600 MHz) for crellasterone B (**2**) in $CDCl_3$

Position	δ_C, type	δ_H (*J* in Hz)	COSY	HMBC	ROESY
1	46.8, CH_2	α 2.21, d (18.8)		2,3,9,10,19	9,11α
		β 2.15, d (18.8)		2,3,9,10,19	19
2	201.1, C				
3	154.3, C				
5	143.6, C				
6	21.9, CH_2	α 2.81, m	6β,7α,7β	3,5,7,8,10	6β,7α,7β
		β 2.12, m	6α,7α	3,5,7	6α,7α
7	31.2, CH_2	α 1.87 m	6α,6β,7β	3,6,8,9	6α,6β,7β,15α
		β 0.95, m	6α,6β,7α	6,8	7α
8	35.4, CH	1.48, m	9	7,9,14	18,19
9	53.4, CH	0.86, m	8,11α	6,12	1α,14
10	40.1, C				
11	23.3, CH_2	α 1.37, m	9,11β	9,12,13	1α,11β,12α,12β
		β 1.52, m	11α,12α,12β	9,12,13	11α,18,19
12	39.0, CH_2	α 1.97, m	11β,12β	14,17	11α,12β,21
		β 1.16, m	12α,11β	13,14,17,18	11α,12α
13	42.4, C				
14	55.4, CH	1.12, m		13,16	9,15α,16α
15	23.8, CH_2	α 1.55, m	15β,16α	12,16	7α,14,15β
		β 1.07, m	15α	8,16,17	15α,16α
16	28.3, CH_2	α 1.67, m	15α,16β	13,14	14,15β,16β,17
		β 1.20, m	16α		16α,20
17	55.3, CH	0.99, m		13,15,18,21,22	16α
18	11.8, CH_3	0.71, s		12,13,14	8,11β,20
19	19.8, CH_3	1.13, s		1,3,9,10	1β,8,11β
20	39.8, CH	1.99, m	21,22	17,21,22,23	22
21	20.5, CH_3	0.97, d (6.6)	20	17,20,22	12α,22
22	135.4, CH	5.13, dd (7.5, 15.2)	20	17,20,21,23,24	20,21
23	131.5, CH	5.14, dd (7.8, 15.2)	24	20,22,24,25,28	24,27,28
24	42.6, CH	1.81, m	23,28	22,23,28,25	23,25,27,28
25	32.7, CH	1.43, m	26,27	23,24,27	24
26	19.2, CH_3	0.81, d (6.8)	25	24,25,27	
27	19.7, CH_3	0.82, d (6.8)	25	26	23,24
28	17.6, CH_3	0.89, d (6.8)	24	23,24,25	23,24

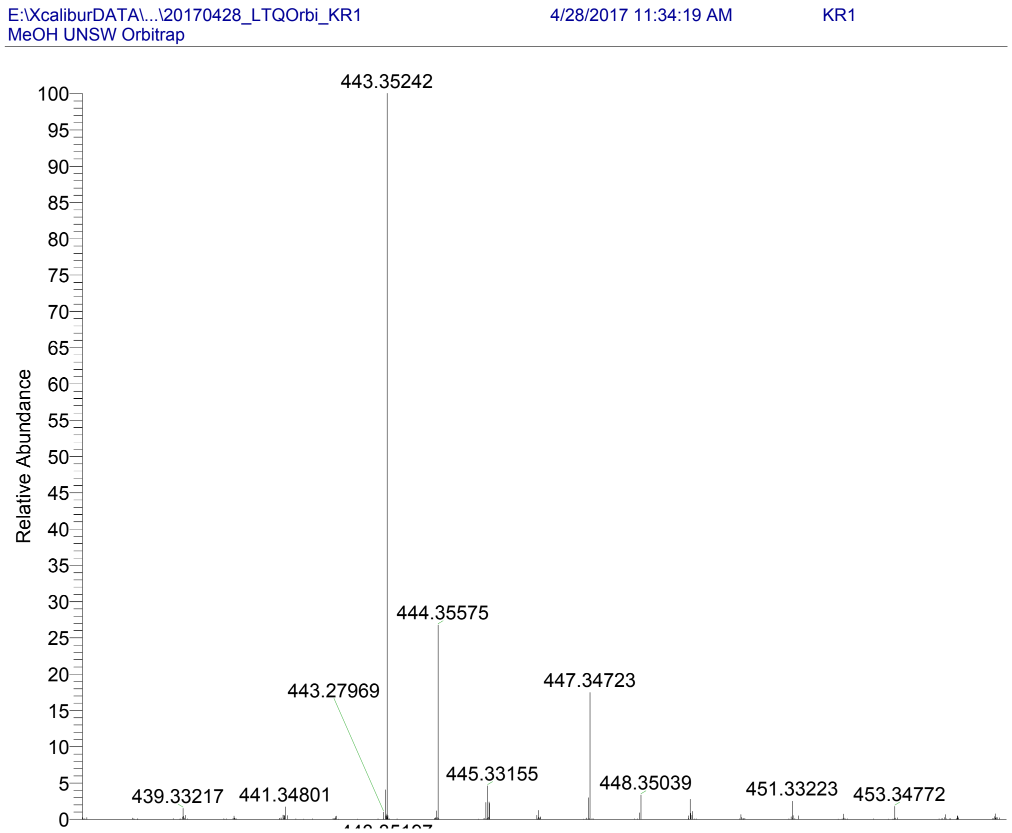

Figure S1. High resolution mass spectrum of crellasterone A (**1**)

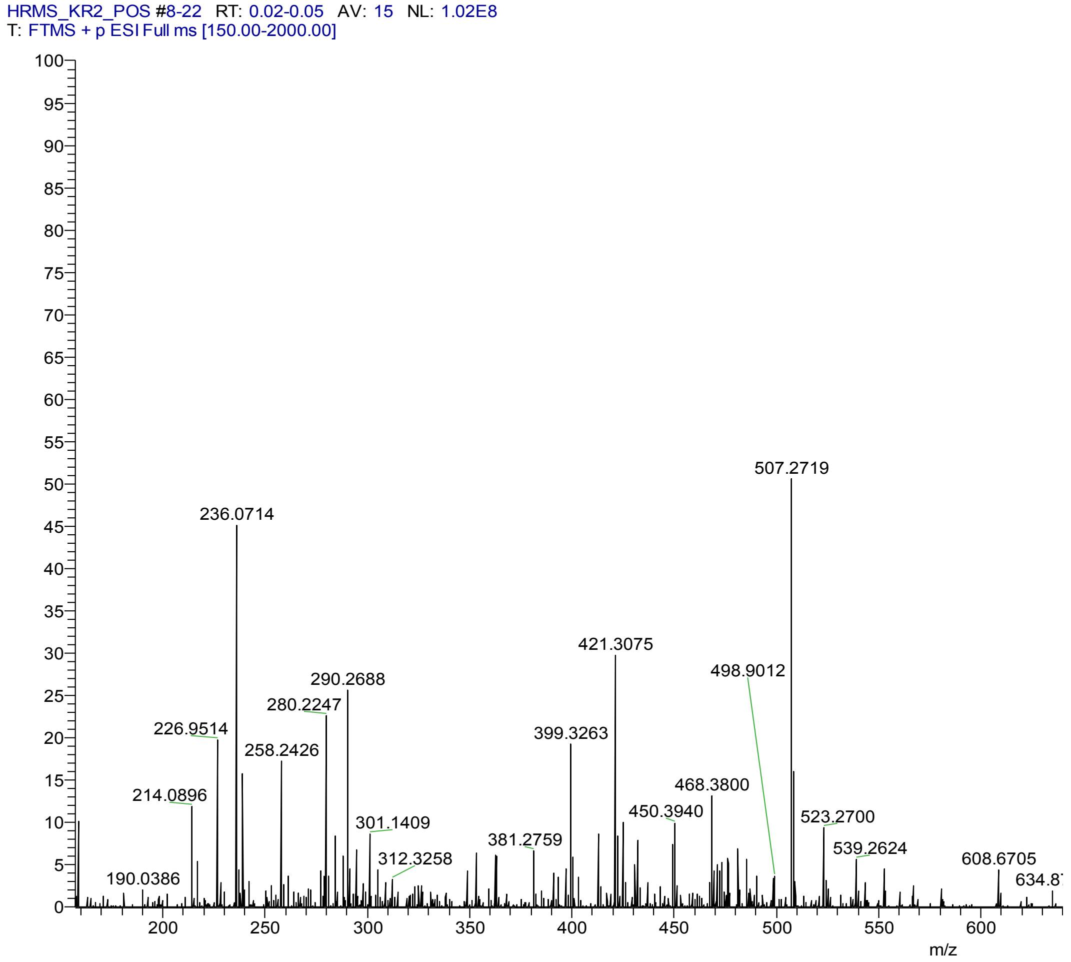

Figure S2. High resolution mass spectrum of crellasterone B (**2**)

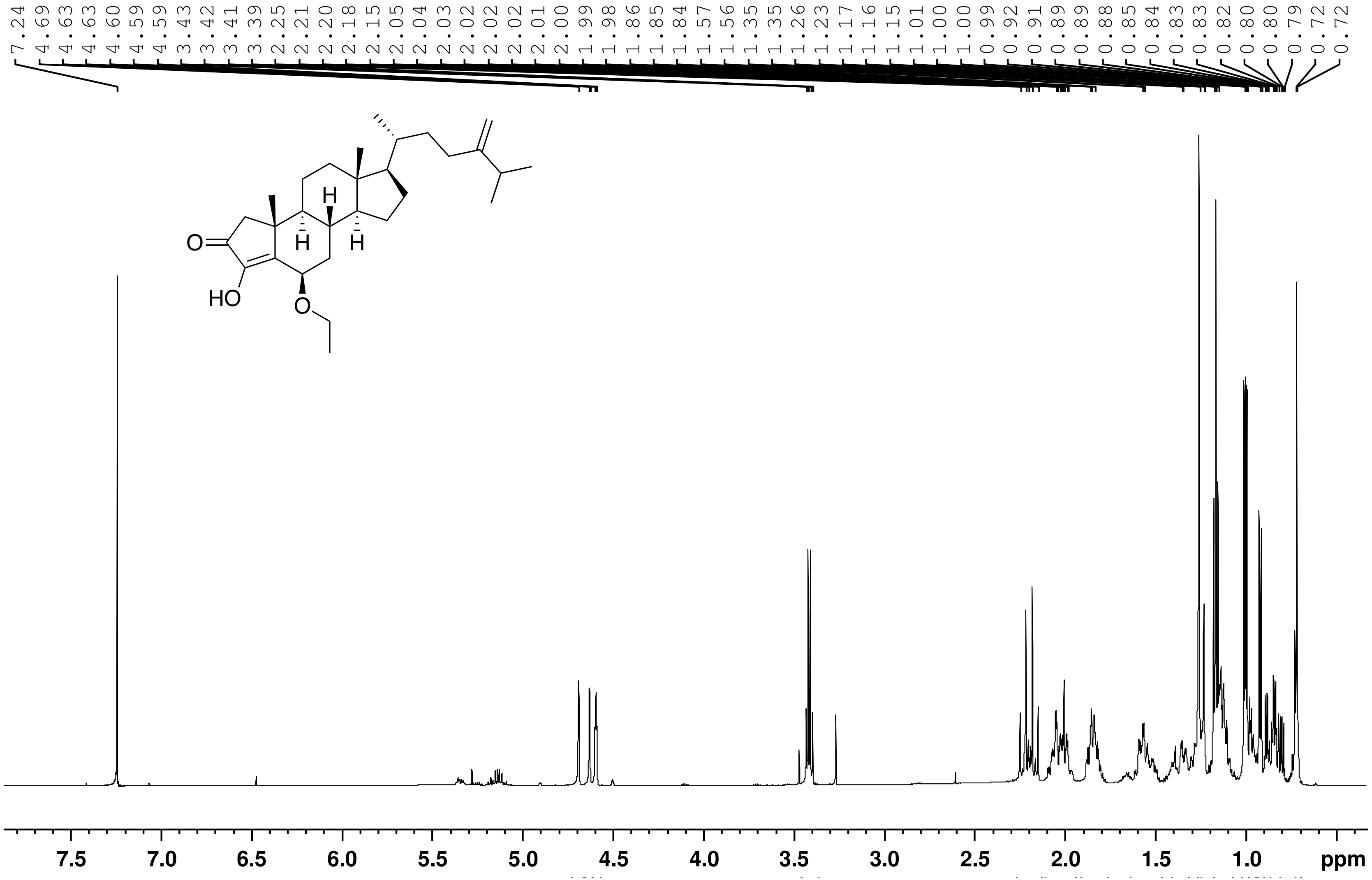
7.24 4.69 4.63 4.63 4.60 4.59 4.59 3.43 3.42 3.41 3.39 2.25 2.21 2.20 2.18 2.15 2.05 2.04 2.03 2.02 2.02 2.02 2.01 2.00 1.99 1.98 1.86 1.85 1.84 1.57 1.56 1.35 1.35 1.26 1.23 1.17 1.16 1.15 1.01 1.00 0.99 0.92 0.91 0.89 0.89 0.88 0.85 0.84 0.83 0.83 0.82 0.80 0.80 0.79 0.72 0.72
O
HO
H
7.5 7.0 6.5 6.0 5.5 5.0 4.5 4.0 3.5 3.0 2.5 2.0 1.5 1.0 ppm

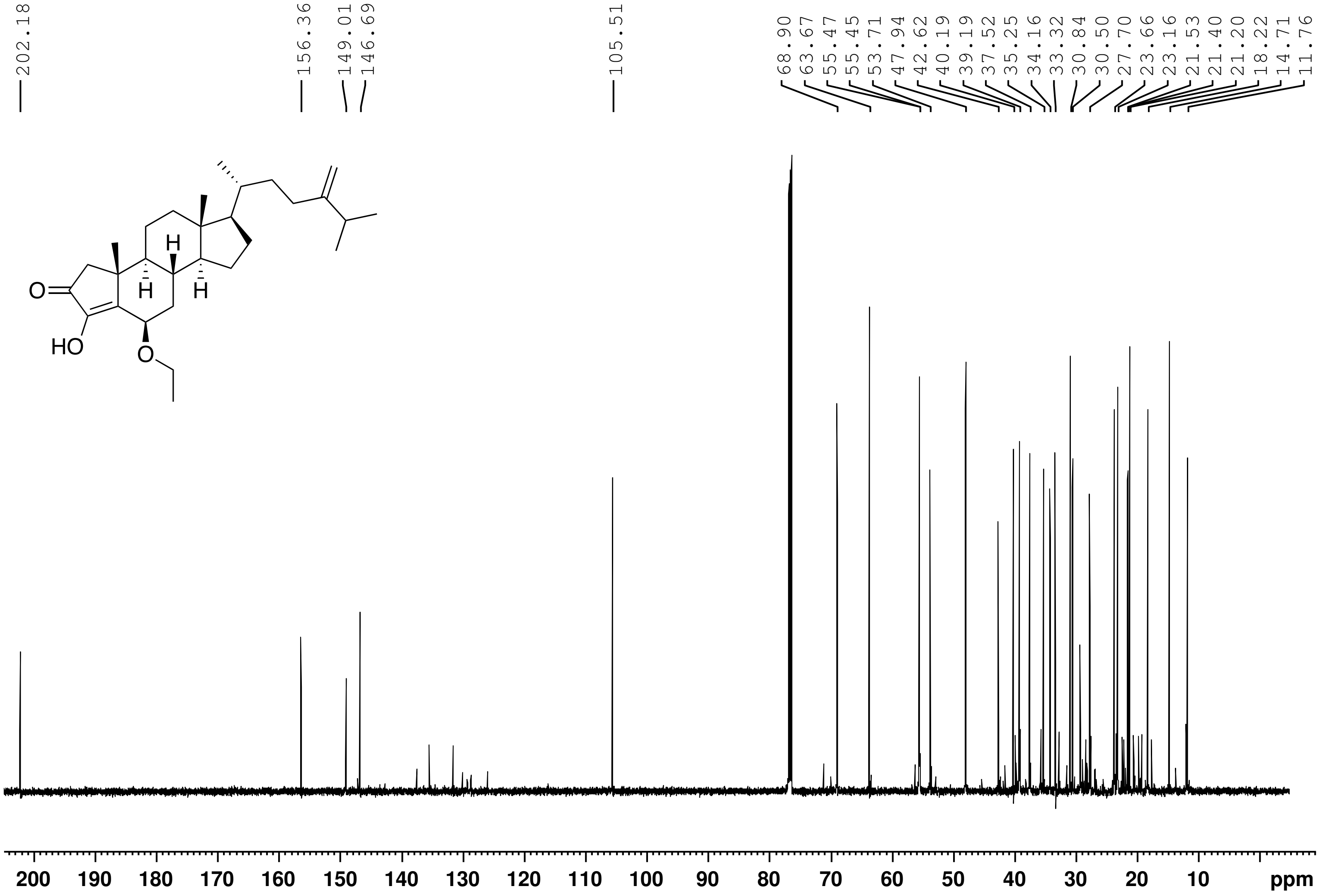

Figure S4. ^{13}C NMR spectrum (150 MHz) of crellasterone A (**1**) in $CDCl_3$

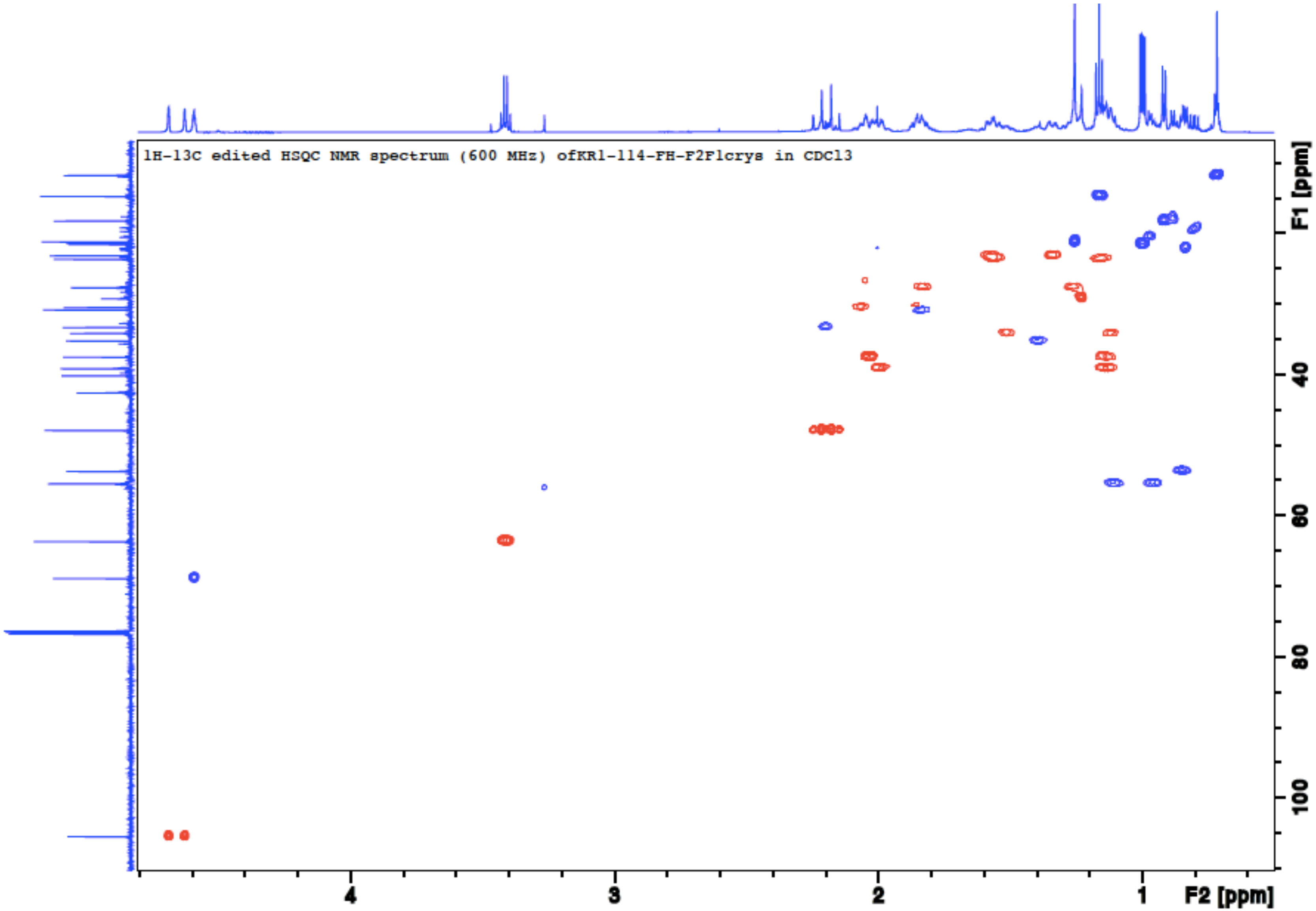

Figure S5. ^{1}H-^{13}C HSQC spectrum (600 MHz) of crellasterone A (**1**) in $CDCl_3$

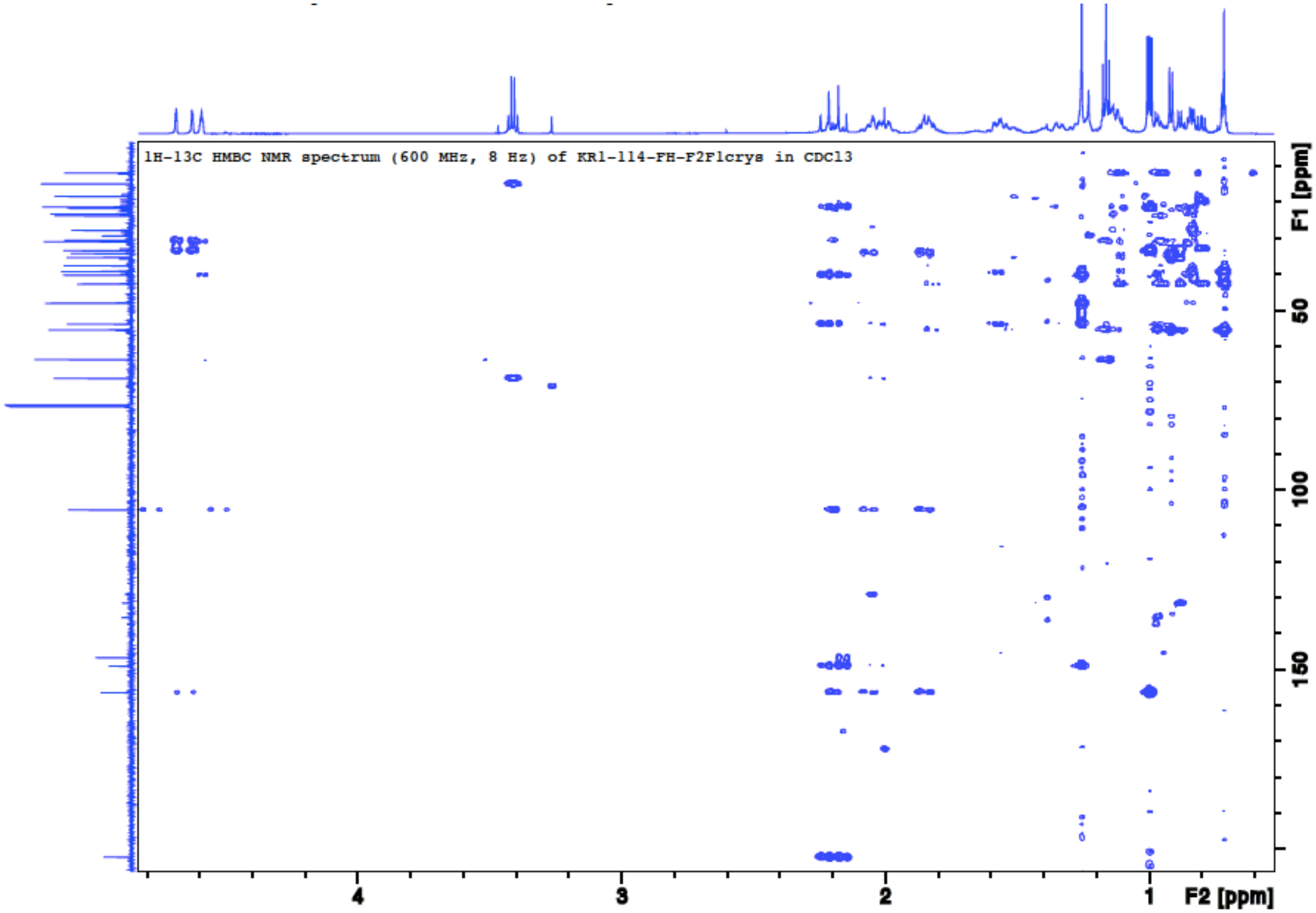

Figure S6. ^{1}H-^{13}C HMBC spectrum (600 MHz) of crellasterone A (**1**) in $CDCl_3$

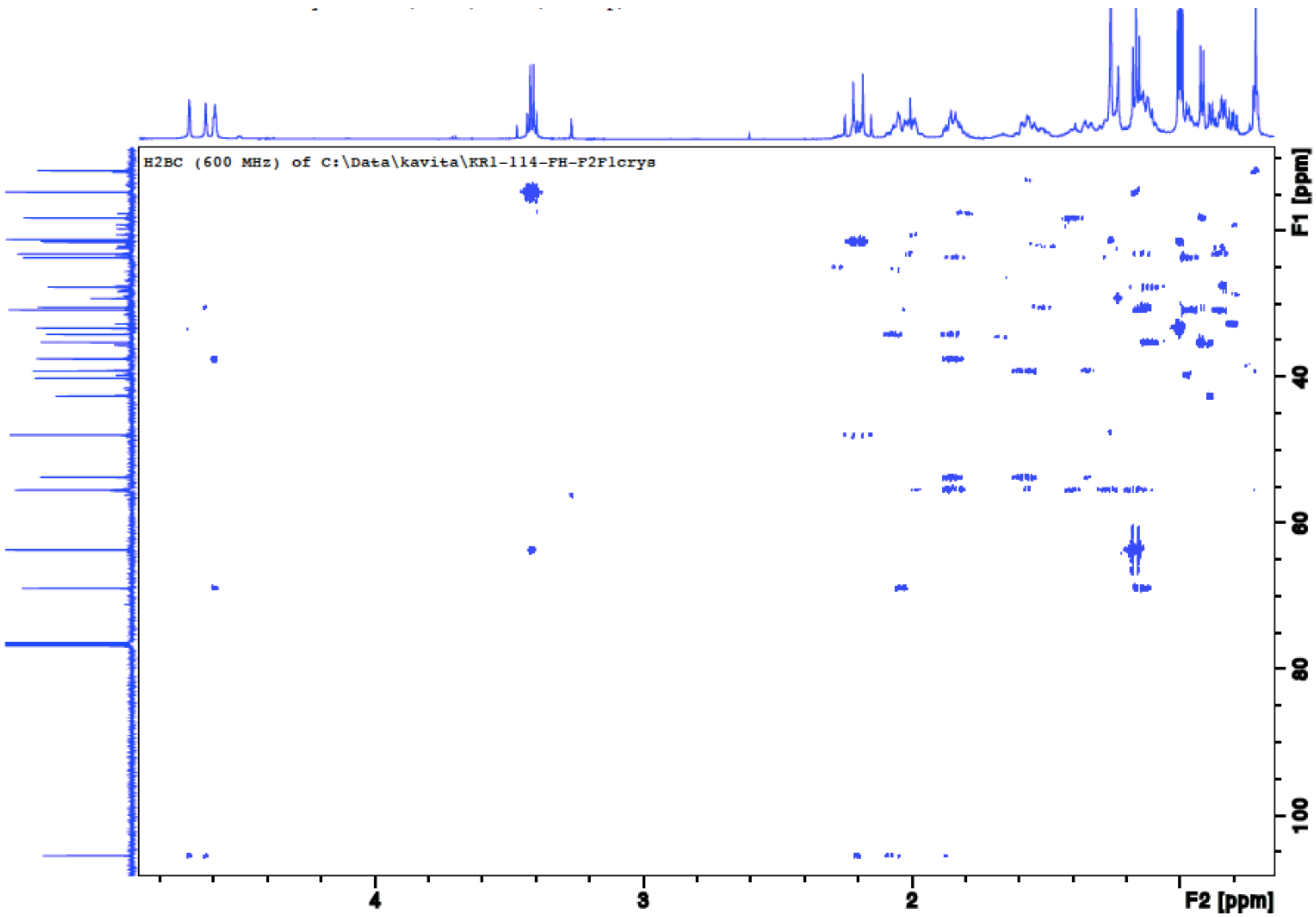

Figure S7. ^{1}H-^{13}C H2BC spectrum (600 MHz) of crellasterone A (**1**) in $CDCl_3$

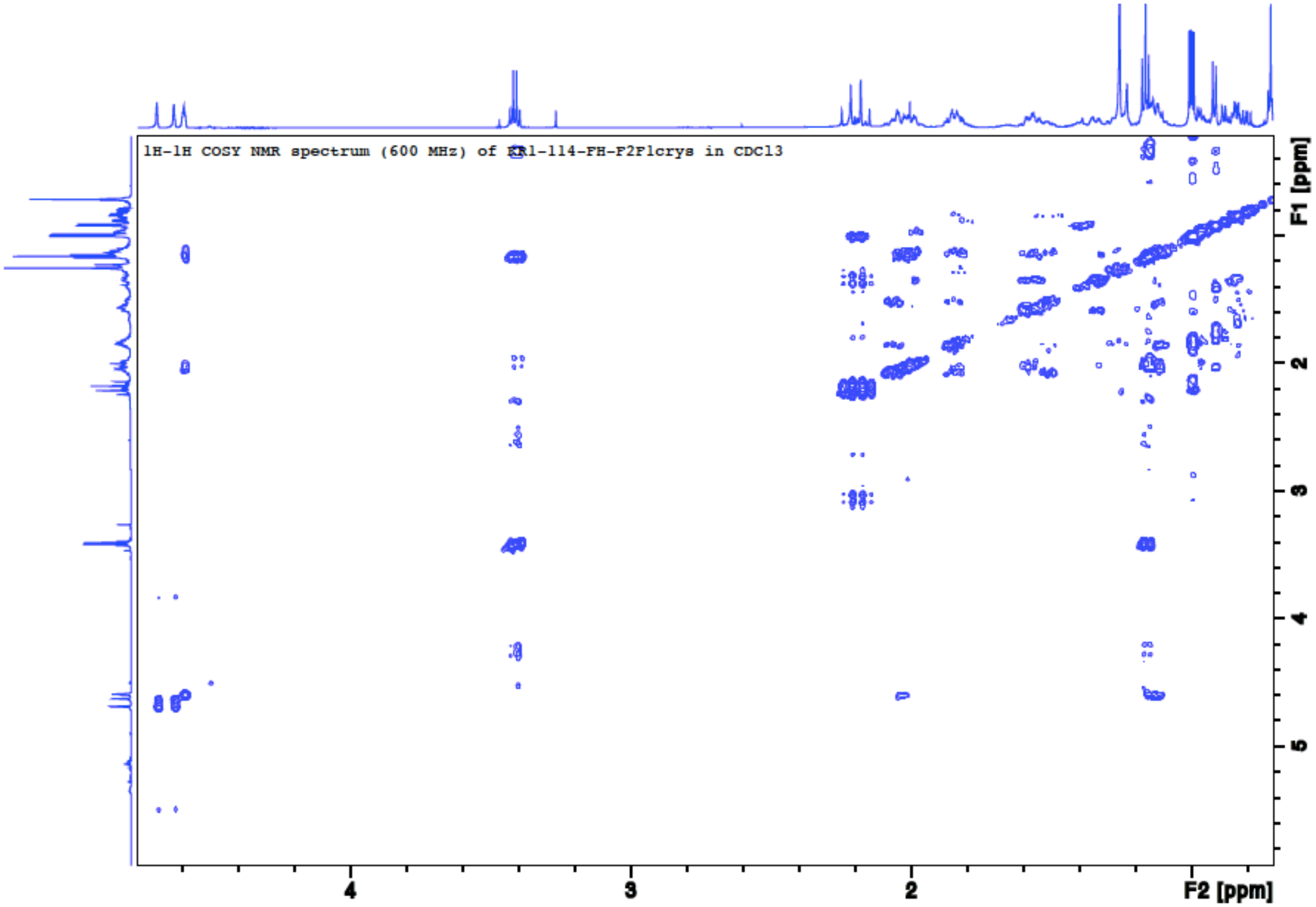

Figure S8. ^{1}H-^{1}H COSY spectrum (600 MHz) of crellasterone A (**1**) in $CDCl_3$

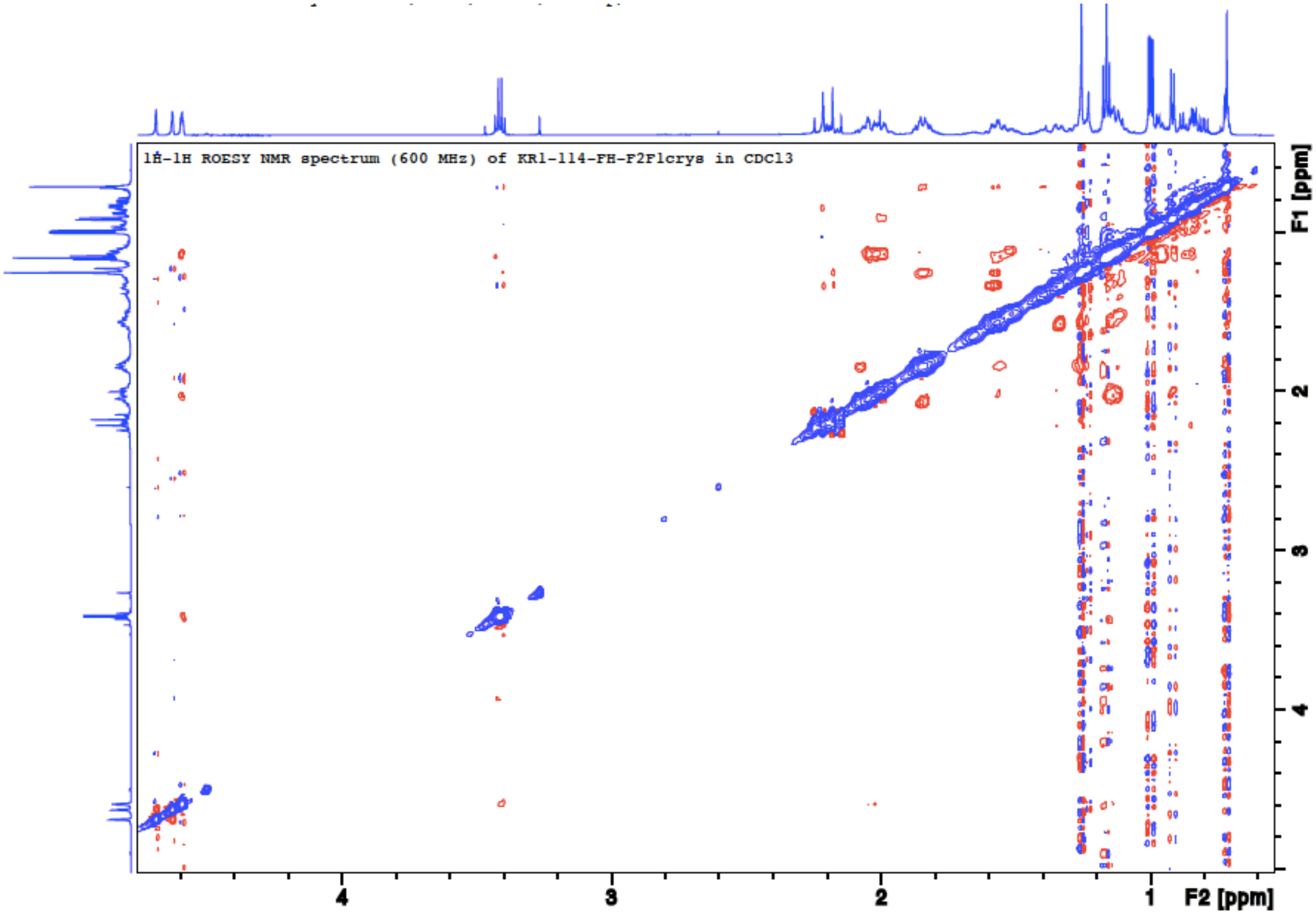

Figure S9. ^{1}H-^{1}H ROESY spectrum (600 MHz) of crellasterone A (**1**) in $CDCl_3$

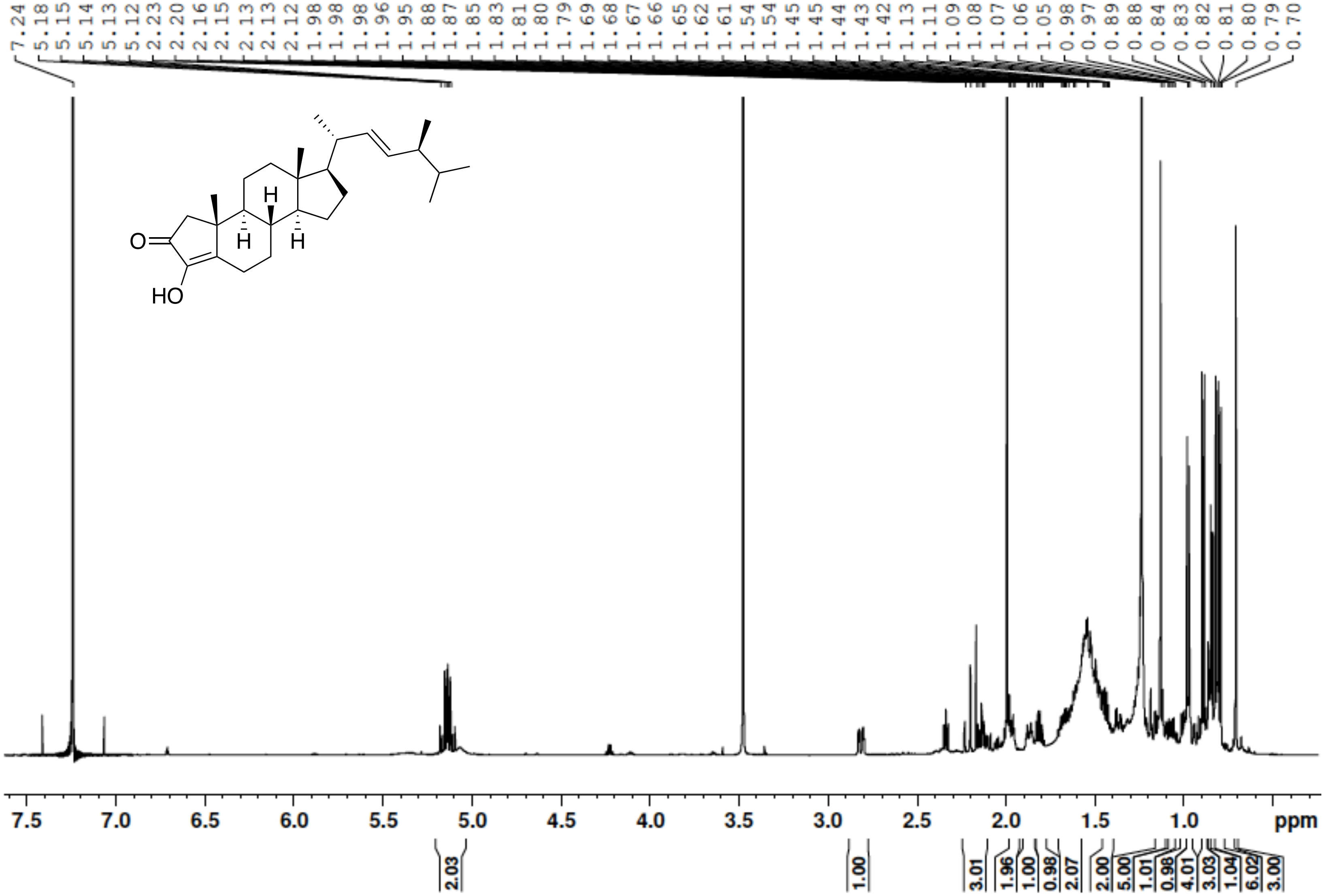

Figure S10. ^{1}H NMR spectrum (600 MHz) of crellasterone B (**2**) in $CDCl_3$

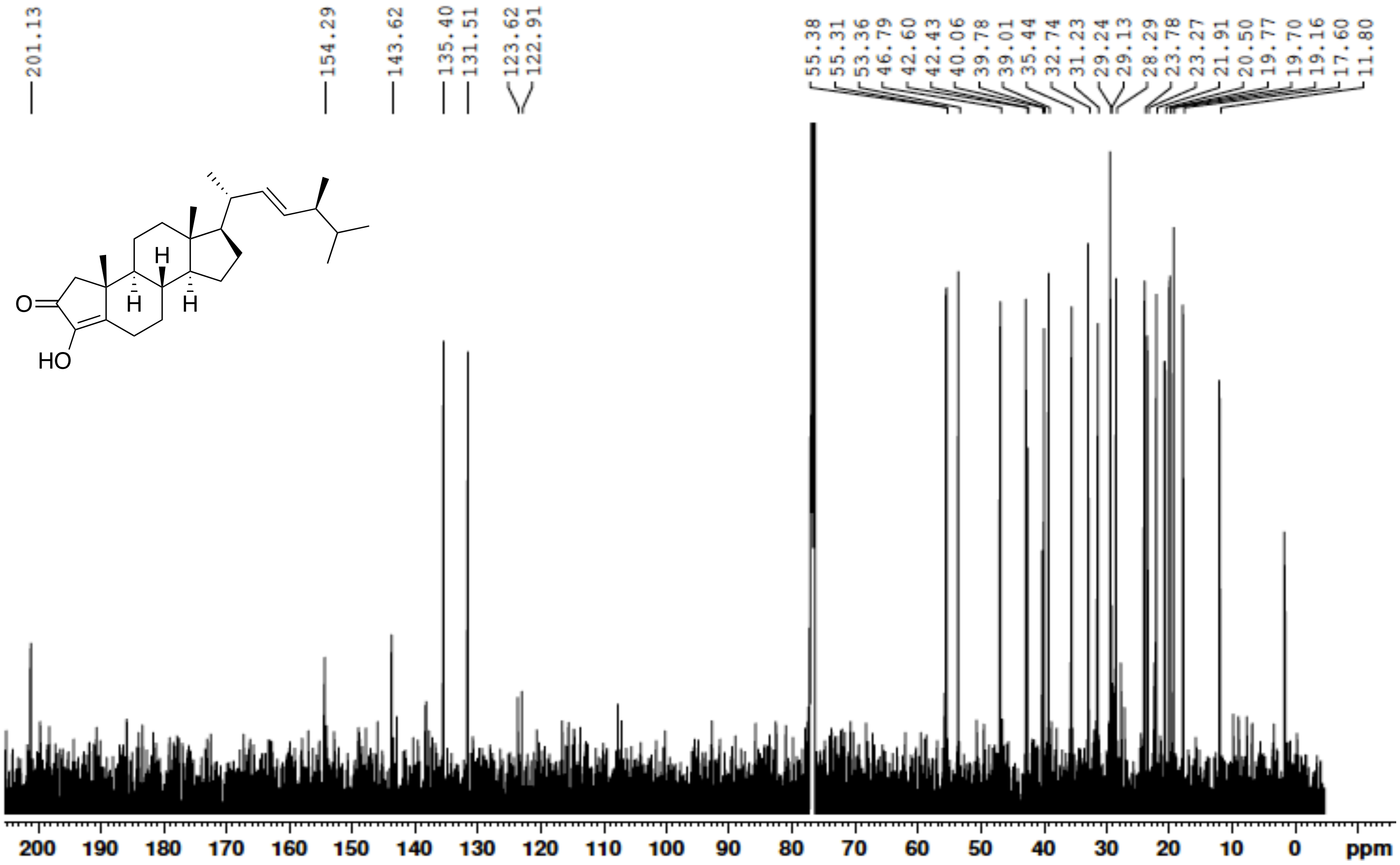

Figure S11. ^{13}C NMR spectrum (150 MHz) of crellasterone B (**2**) in $CDCl_3$

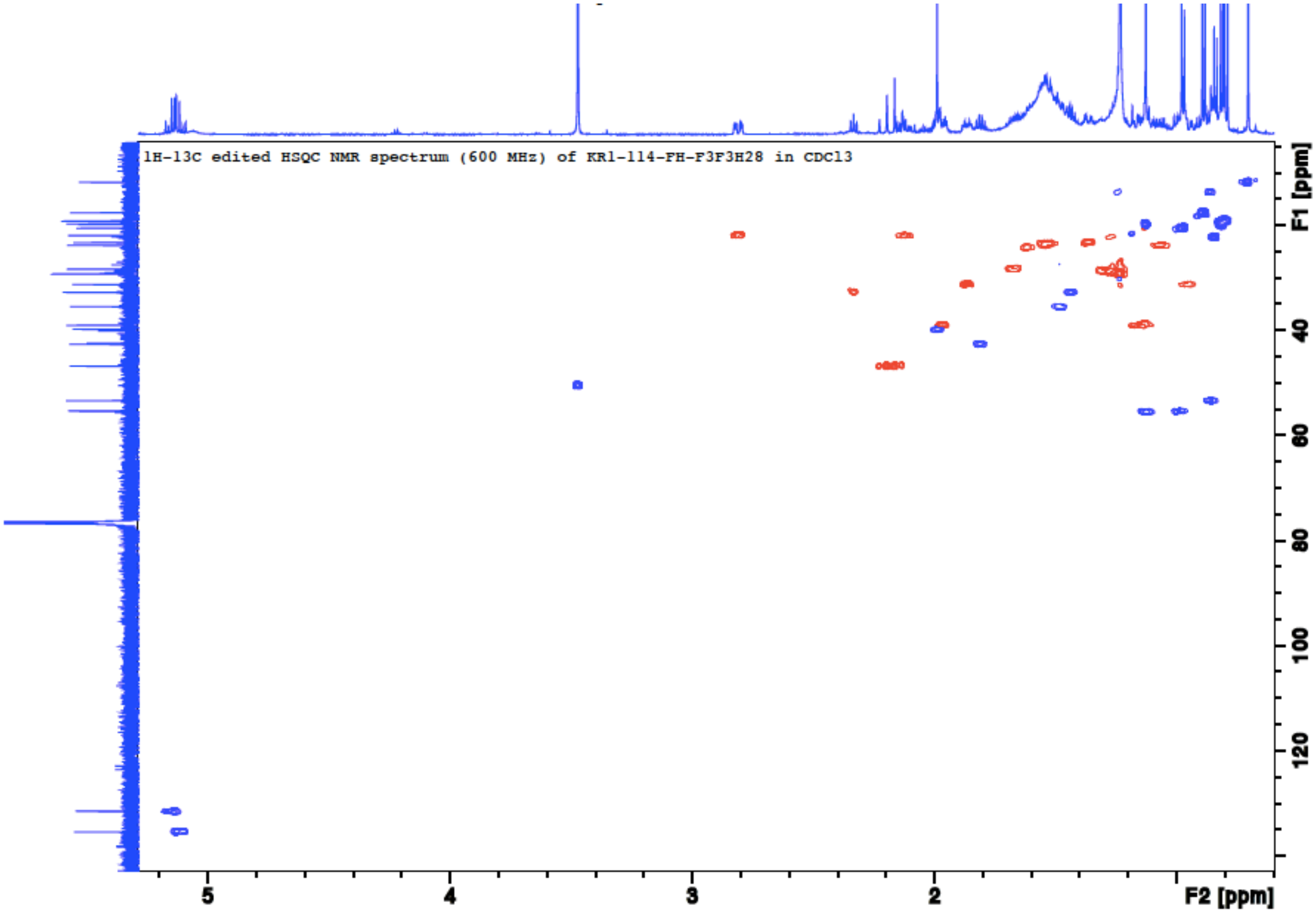

Figure S12. ^{1}H-^{13}C HSQC spectrum (600 MHz) of crellasterone B (**2**) in $CDCl_3$

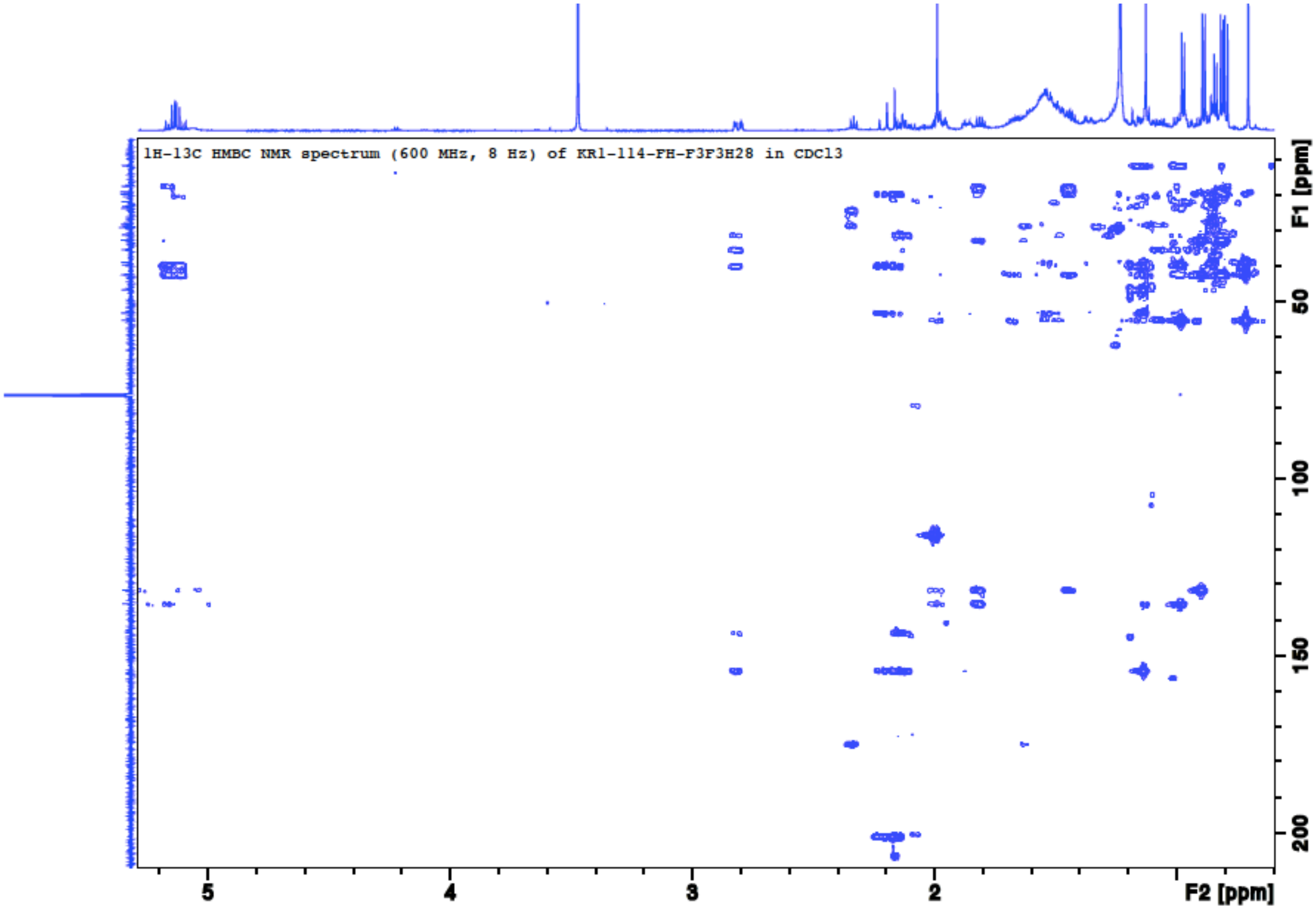

Figure S13. ^{1}H-^{13}C HMBC spectrum (600 MHz) of crellasterone B (**2**) in $CDCl_3$

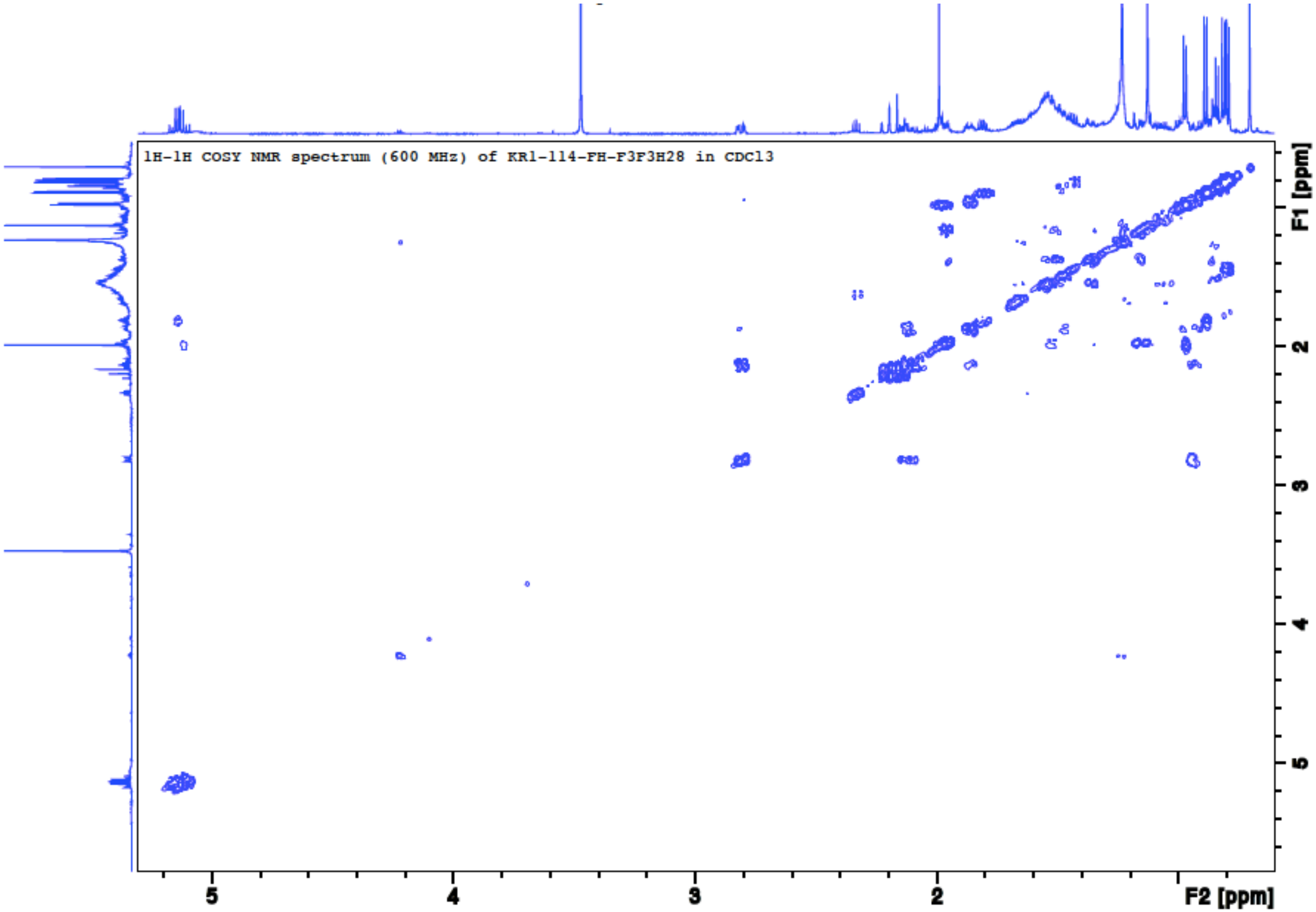

Figure S14. ^{1}H-^{1}H COSY spectrum (600 MHz) of crellasterone B (**2**) in $CDCl_3$

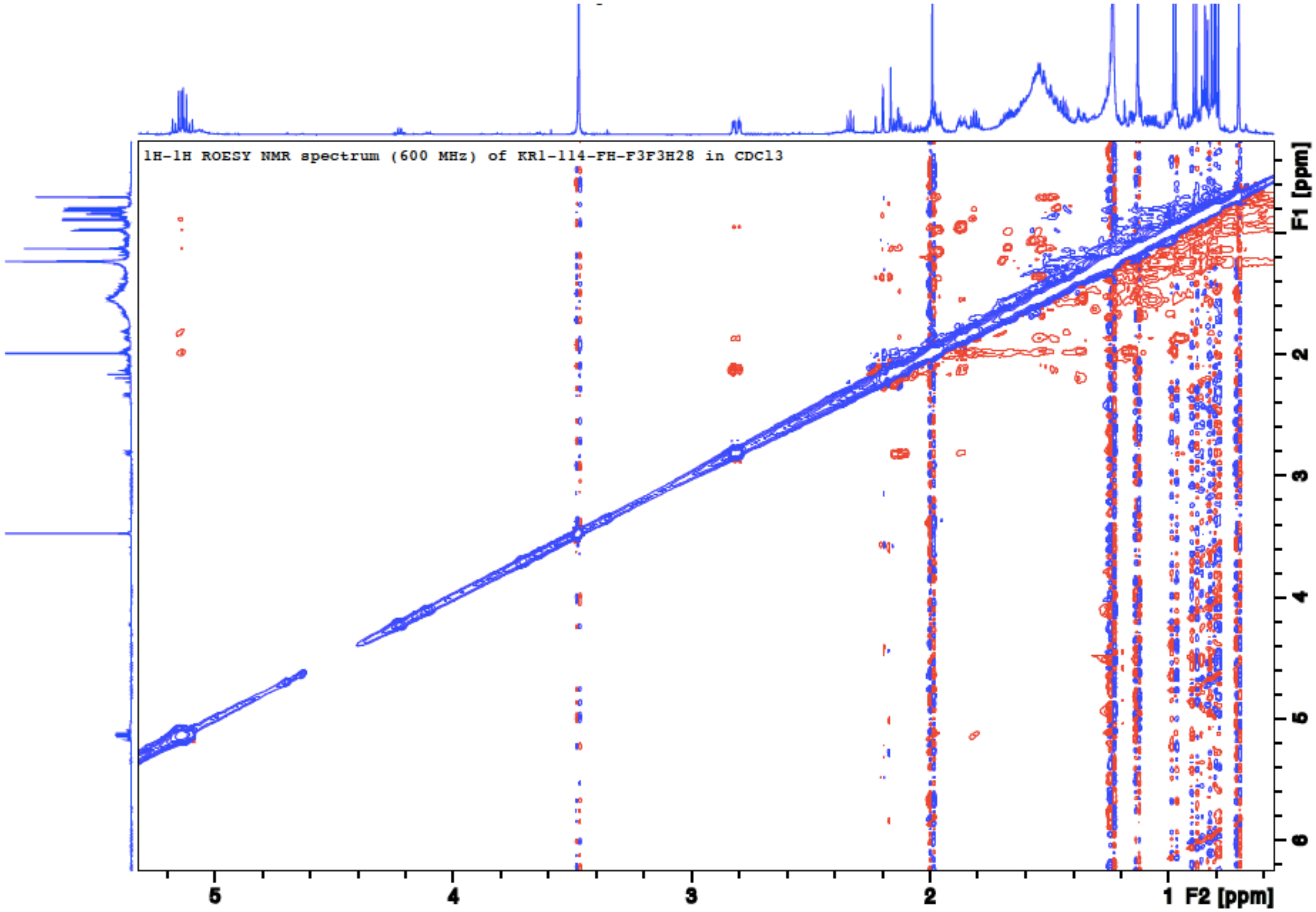

Figure S15. ^{1}H-^{1}H ROESY spectrum (600 MHz) of crellasterone B (**2**) in $CDCl_3$

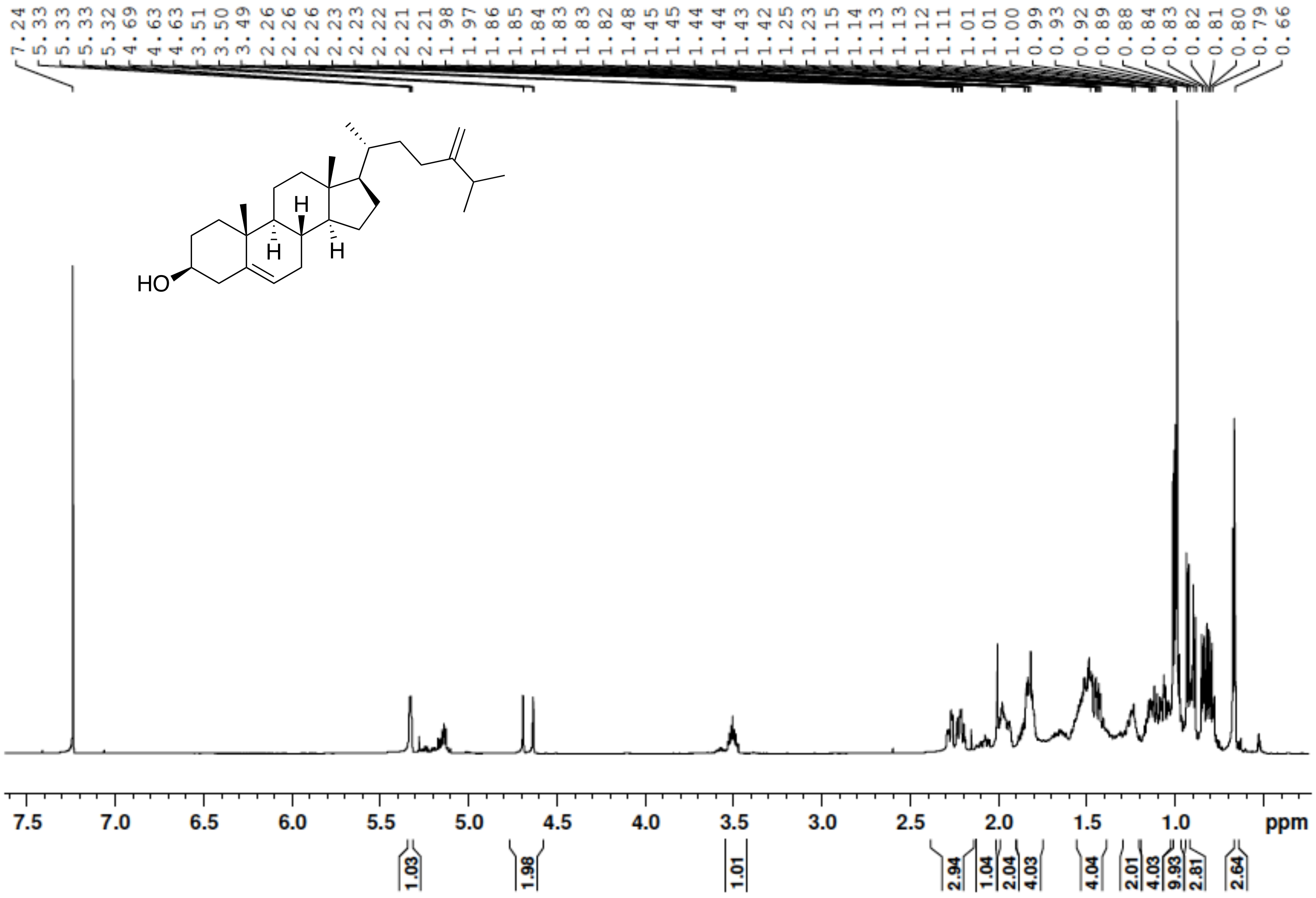

Figure S16. ^{1}H NMR spectrum (600 MHz) of chalinasterol (**3**) in $CDCl_3$

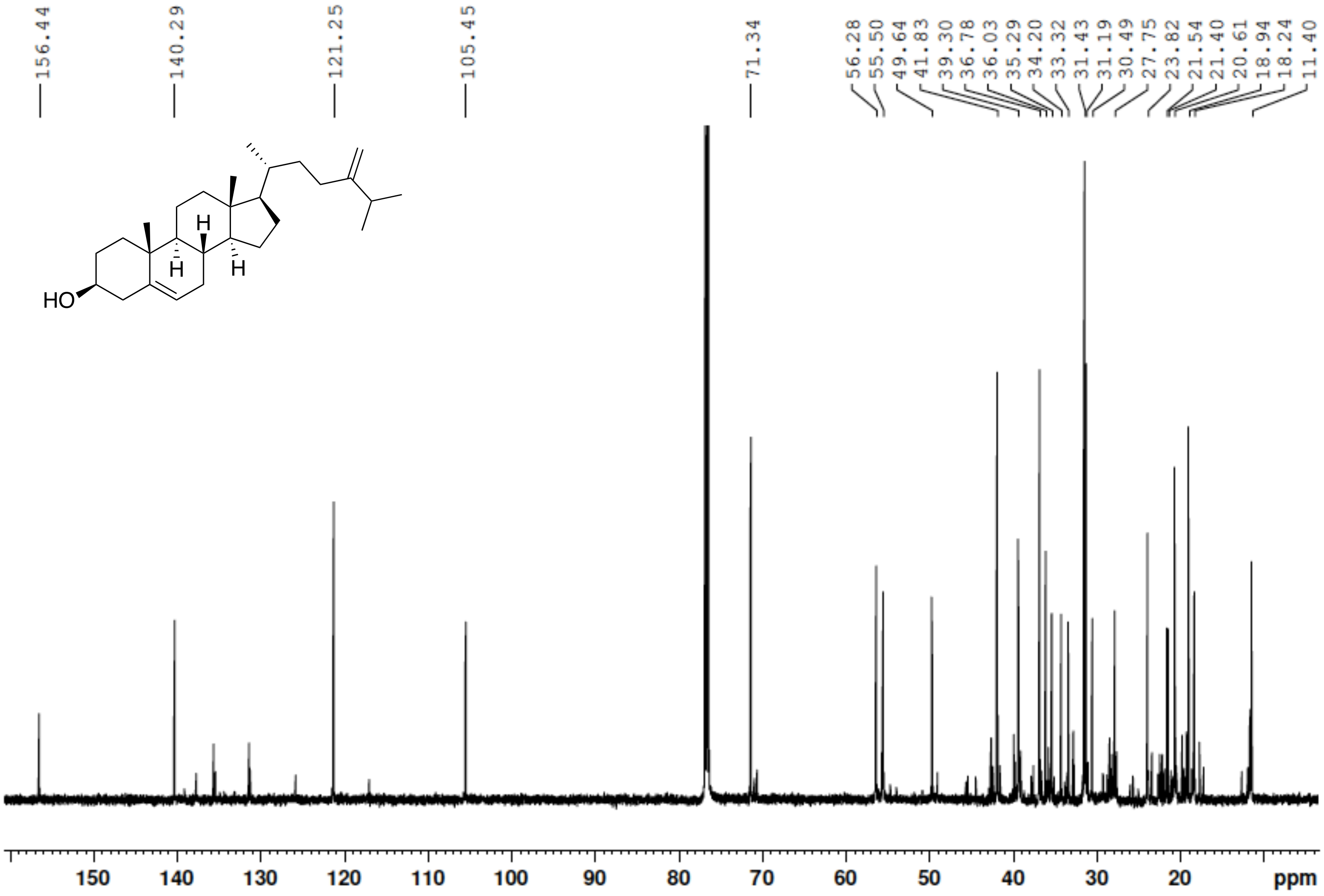

Figure S17. ^{13}C NMR spectrum (150 MHz) of chalinasterol (**3**) in $CDCl_3$

TD-DFT calculation (Turbomole 7.1) of 1 and 2:

Figure S18.Coordinate files for crellasterone B (**2**; minus sidechain) (XYZ format) from Turbomole DFT-D3//PBE0/TZVPP-COSMO ($CHCl_3$) calculations

```
49

C -1.762526 -0.476260 0.660610
O -1.821955 0.620006 1.437198
H -2.566125 0.491023 2.041342
C -2.719927 -1.558425 0.831002
O -3.630028 -1.539528 1.641838
C -2.341500 -2.657841 -0.120898
H -2.040510 -3.529493 0.466719
H -3.203150 -2.959478 -0.719062
C -1.174904 -2.097820 -0.956511
C -1.617660 -1.885110 -2.407803
H -2.475451 -1.210053 -2.438994
H -1.915885 -2.827542 -2.868130
H -0.825496 -1.447606 -3.016512
C -0.882071 -0.762845 -0.309868
C 0.097754 -2.968079 -0.848778
H 0.259293 -3.126076 0.228456
C 0.317046 -0.000412 -0.722767
H 0.436327 0.897481 -0.114838
H 0.204187 0.325735 -1.763441
C 1.544134 -0.908927 -0.618161
H 1.744510 -1.118288 0.438975
H 2.419281 -0.386054 -1.010158
C 1.350530 -2.229323 -1.353165
H 1.232728 -2.006925 -2.420751
C 2.544145 -3.148595 -1.173425
H 2.579185 -3.396421 -0.101592
C 2.409134 -4.495217 -1.896728
C 2.296281 -4.336009 -3.413160
H 3.190114 -3.880529 -3.842491
H 1.446177 -3.718459 -3.701791
H 2.164422 -5.311722 -3.885472
C 1.185409 -5.212007 -1.344665
H 1.356797 -5.431364 -0.284067
H 1.031547 -6.171713 -1.848006
C -0.063888 -4.346527 -1.487357
H -0.918455 -4.858597 -1.036260
H -0.304866 -4.235741 -2.547201
C 3.938079 -2.661283 -1.558865
H 4.357231 -1.979465 -0.817844
H 3.904970 -2.117565 -2.507099
C 3.762391 -5.126347 -1.527756
C 4.156827 -6.387132 -2.270392
H 4.292082 -6.196635 -3.337424
H 3.403141 -7.170994 -2.159878
H 5.100726 -6.781391 -1.886358
H 3.700150 -5.368952 -0.459673
C 4.760552 -3.961709 -1.695322
H 5.559600 -4.021771 -0.954264
H 5.241644 -4.018014 -2.675210
```

CD spectrum TD-DFT-D3//PBE0/TZVPP

wavelength	rotary strength
203.7521581	2.255092219
217.8472135	19.68469916
260.4504212	16.21513188
291.7136443	-17.45394418

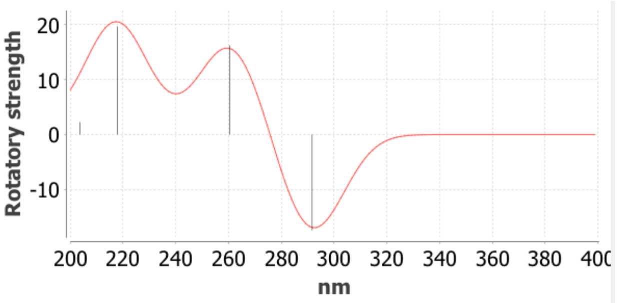

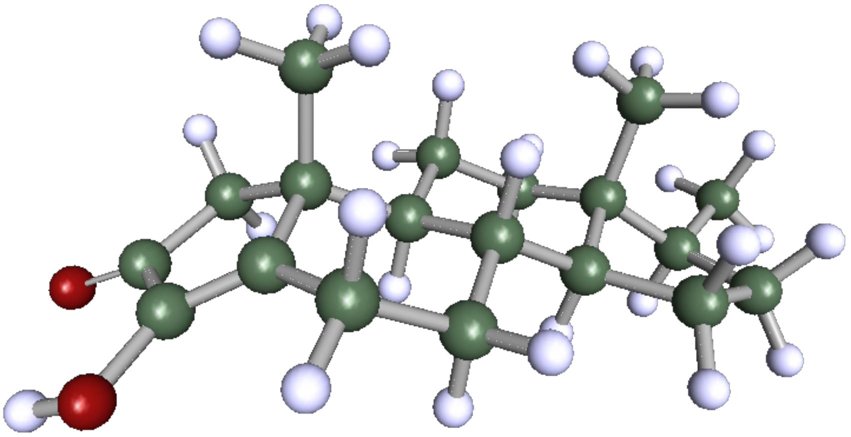

Figure S19.Coordinate files for 6α-crellasterone A
(**2a**; minus sidechain) (XYZ format) from Turbomole DFT-D3//PBE0/TZVPP-COSMO ($CHCl_3$) calculations

```
56

C 1.670922 0.649083 0.493113
O 2.077400 1.824824 -0.008693
H 1.565328 1.958646 -0.823692
C 2.278062 0.121140 1.719909
O 3.122621 0.670216 2.395622
C 1.679023 -1.242120 1.961415
H 2.474786 -1.982177 1.838276
H 1.324357 -1.321738 2.990087
C 0.571176 -1.420133 0.910622
C -0.805817 -1.397708 1.583781
H -0.906470 -2.218431 2.294481
H -1.619042 -1.477576 0.860639
H -0.934752 -0.461840 2.131155
C 0.739859 -0.203709 0.034442
C 0.780574 -2.674258 0.034958
H 1.825509 -2.626792 -0.305919
C -0.039766 -0.105648 -1.223543
O 0.345028 1.071054 -1.915971
C -0.610131 1.544577 -2.849806
C -0.099254 2.829459 -3.446117
H 0.046163 3.585488 -2.672487
H -0.817386 3.215094 -4.171325
H 0.851123 2.667446 -3.957226
H -1.566642 1.703041 -2.336093
H -0.770441 0.794731 -3.631768
H -1.111103 -0.037190 -0.978383
C 0.195135 -1.368933 -2.047940
H 1.238437 -1.362102 -2.381743
H -0.435955 -1.351215 -2.938344
C -0.074894 -2.635358 -1.243449
H -1.137098 -2.645529 -0.971048
C 0.233227 -3.879660 -2.055154
H 1.316901 -3.858950 -2.244748
C -0.022532 -5.191554 -1.300379
C -1.489931 -5.364366 -0.908565
H -1.622364 -6.289727 -0.344421
H -2.141343 -5.415041 -1.782426
H -1.848878 -4.549143 -0.281153
C 0.864168 -5.212128 -0.063020
H 1.912581 -5.224784 -0.383307
H 0.698178 -6.120937 0.523316
C 0.615861 -3.982081 0.806692
H 1.301535 -3.989164 1.658571
H -0.389882 -4.037055 1.229546
C -0.447407 -4.090829 -3.404160
H -0.005841 -3.477358 -4.190814
H -1.505043 -3.819919 -3.345449
C 0.358105 -6.199607 -2.397876
C -0.002565 -7.650646 -2.151243
H 0.365331 -8.282271 -2.963339
H -1.084395 -7.789731 -2.093225
H 0.436360 -8.020913 -1.221380
H 1.448429 -6.135484 -2.500786
C -0.276946 -5.602647 -3.671273
H 0.345121 -5.797309 -4.546507
H -1.243122 -6.075337 -3.866253
```

CD spectrum TD-DFT-D3//PBE0/TZVPP

wavelength	rotary strength
206.662851916	7.926936143424
216.412430933	-8.916505274895
262.157607884	41.256310578241
304.982195300	-25.2656903404

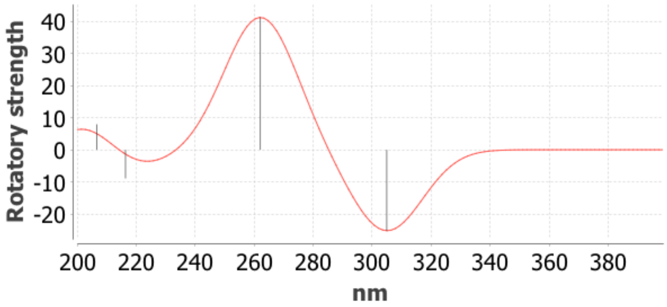

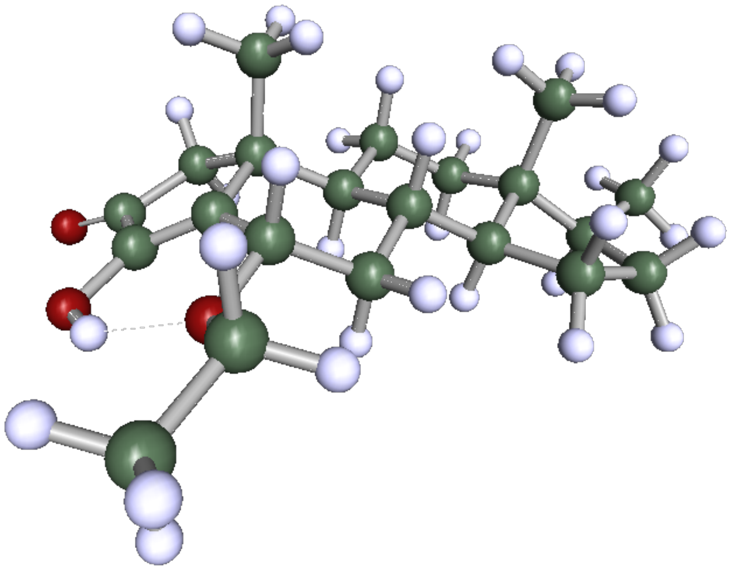

Figure S20.Coordinate files for 6α-crellasterone A
(**2a**; minus sidechain) (XYZ format) from Turbomole DFT-D3//PBE0/TZVPP-COSMO ($CHCl_3$) calculations

```
56

C 1.985152 0.656455 -0.688838
O 2.319321 1.617957 -1.563779
H 3.168420 1.969994 -1.257804
C 2.892441 0.362454 0.413973
O 3.935873 0.964048 0.597827
C 2.320681 -0.778058 1.199461
H 3.014675 -1.620687 1.146139
H 2.233159 -0.504656 2.252579
C 0.967034 -1.095413 0.538895
C -0.178612 -0.813418 1.515982
H -1.155017 -1.021655 1.077114
H -0.162573 0.236632 1.814779
H -0.079091 -1.419725 2.416705
C 0.904359 -0.139635 -0.638097
C 0.928035 -2.535908 -0.020870
H 1.846360 -2.645329 -0.616859
C -0.228443 -0.293858 -1.594472
O -0.197978 0.596534 -2.678129
C -0.665302 1.891958 -2.353380
C -0.500094 2.777583 -3.559632
H -1.053702 2.379182 -4.411761
H 0.553339 2.852584 -3.832973
H -0.873900 3.780127 -3.344301
H -0.099763 2.295138 -1.505963
H -1.720366 1.829587 -2.052040
H -1.171995 -0.159067 -1.041032
C -0.203099 -1.720596 -2.127533
H 0.705306 -1.850312 -2.725612
H -1.057683 -1.854052 -2.793635
C -0.235101 -2.746577 -1.003661
H -1.184789 -2.629261 -0.468377
C -0.149806 -4.164183 -1.537193
H 0.846655 -4.259073 -1.994087
C -0.201369 -5.240571 -0.444234
C -1.516982 -5.221388 0.334310
H -1.701099 -4.259574 0.811905
H -1.498676 -5.976615 1.123150
H -2.372942 -5.435761 -0.307150
C 0.975737 -5.022587 0.495825
H 1.905262 -5.179422 -0.063999
H 0.967714 -5.751876 1.312126
C 0.963556 -3.606868 1.067565
H 1.844677 -3.459260 1.697585
H 0.099333 -3.490894 1.725697
C -1.159030 -4.644229 -2.575593
H -0.939438 -4.261488 -3.573069
H -2.165141 -4.301750 -2.318467
C -0.120290 -6.502688 -1.319450
C -0.407684 -7.827581 -0.642252
H 0.253888 -7.994297 0.211573
H -0.260919 -8.656117 -1.339154
H -1.438937 -7.879066 -0.285500
H 0.906261 -6.536370 -1.704790
C -1.062661 -6.183976 -2.499222
H -0.687366 -6.618190 -3.427362
H -2.046188 -6.628202 -2.324821
```

CD spectrum TD-DFT-D3//PBE0/TZVPP

wavelength	rotary strength
211.424067019	10.376465431112
240.810059529	0.0390457923757
261.074280696	24.797654587273
290.904565365	-22.00899476656

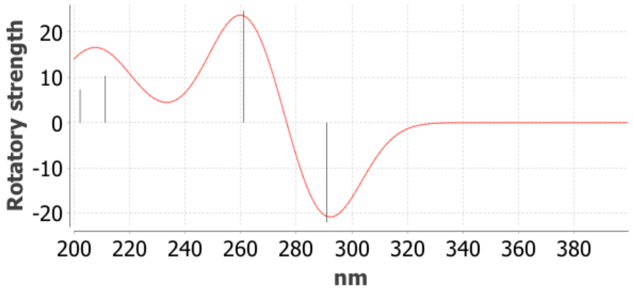

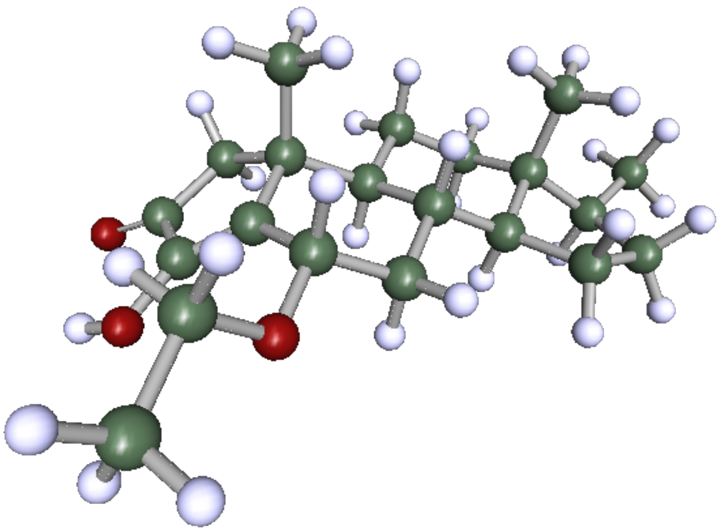

Figure S21.Coordinate files for 6β-crellasterone A
(**2b**; minus sidechain) (XYZ format) from Turbomole DFT-D3//PBE0/TZVPP-COSMO ($CHCl_3$) calculations

```
56

C 1.629755 0.567542 0.642312
O 2.391988 1.584684 0.214823
H 2.854490 1.931425 0.990757
C 1.624434 0.190109 2.053426
O 2.259122 0.776470 2.910490
C 0.759251 -1.028335 2.193248
H 1.401197 -1.864338 2.485574
H 0.028920 -0.892603 2.992440
C 0.127591 -1.254972 0.804519
C -1.378363 -0.975062 0.869640
H -1.864629 -1.641988 1.582412
H -1.852338 -1.092700 -0.103426
H -1.549952 0.050560 1.203425
C 0.826017 -0.235154 -0.069284
C 0.442743 -2.658206 0.239490
H 1.529802 -2.789001 0.354118
C 0.680971 -0.273540 -1.556516
O -0.624539 0.070303 -1.987407
C -0.950167 1.426008 -1.759489
C -2.349818 1.673509 -2.258113
H -2.426778 1.448557 -3.323302
H -2.623512 2.718954 -2.106327
H -3.066396 1.049539 -1.720925
H -0.228296 2.068434 -2.282261
H -0.875974 1.657697 -0.689405
H 1.398735 0.423109 -2.008428
C 0.966483 -1.689427 -2.029590
H 2.036536 -1.884638 -1.899596
H 0.754654 -1.741064 -3.099991
C 0.169856 -2.744185 -1.272091
H -0.893895 -2.565661 -1.460648
C 0.529283 -4.142148 -1.739850
H 1.588267 -4.289230 -1.477399
C -0.227924 -5.258328 -1.008616
C -1.739457 -5.176875 -1.223464
H -2.008943 -5.308561 -2.272599
H -2.151771 -4.221603 -0.899941
H -2.243861 -5.959603 -0.652935
C 0.105012 -5.167213 0.473545
H 1.176345 -5.360949 0.604489
H -0.429694 -5.932833 1.044518
C -0.231486 -3.783316 1.022474
H 0.064364 -3.722705 2.073767
H -1.314851 -3.645320 1.006256
C 0.386064 -4.504942 -3.215460
H 1.197714 -4.100436 -3.821536
H -0.544757 -4.098582 -3.620430
C 0.347569 -6.484764 -1.736806
C -0.340179 -7.812882 -1.492370
H -1.368147 -7.804414 -1.861629
H -0.365289 -8.062567 -0.428529
H 0.185854 -8.620121 -2.007705
H 1.387281 -6.575524 -1.398646
C 0.363334 -6.049535 -3.216953
H 1.222879 -6.472573 -3.739966
H -0.526496 -6.426000 -3.728091
```

CD spectrum TD-DFT-D3//PBE0/TZVPP

wavelength	rotary strength
210.32764955009	-4.8560094925472
222.29418556585	17.796859718369
248.22792078821	38.55729154899
262.73255124129	-15.299318109639
300.78249687242	-20.823666181965

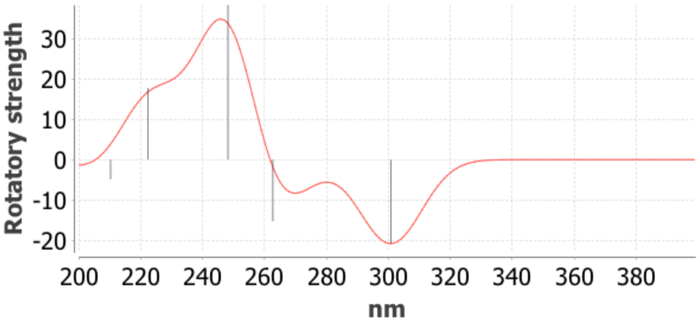

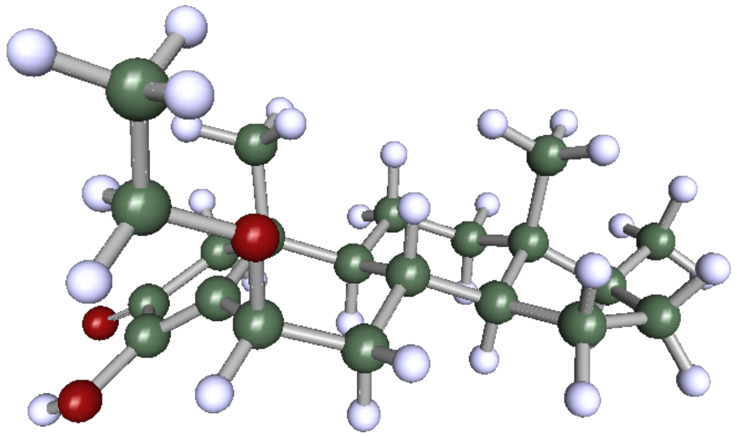

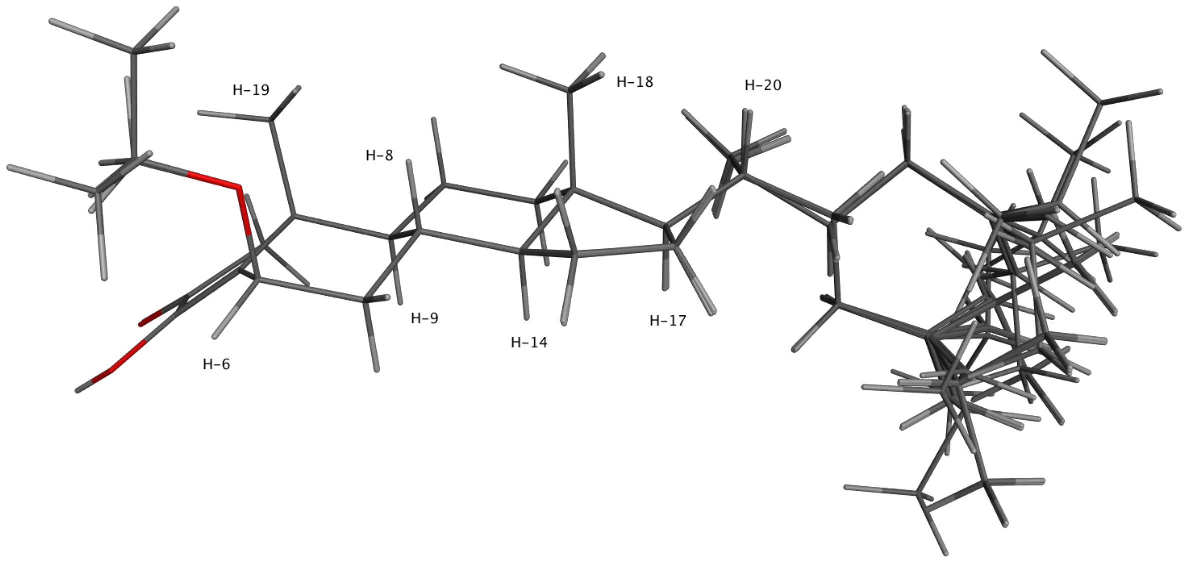

Figure S22. Ten lowest energy structure of **1**, superimposed showing the relative distances between protons.

Calculations were performed in MOE. Low mode molecular dynamics with a RMSD of 0.25 Å and a 7 kcal/mol energy window was used to generate a database of conformations. Structures were minimized to an RMS gradient of <0.001 kcal/mol using the MMFF94x forcefield and ranked by energy. The ten lowest structures were within 1.5 kcal/mol of each other and are shown in Figure S22. All structures show H-20 and H_3-18 in close proximity for (20*R*)-crellasterone A as seen experimentally in the ROESY spectrum (Figure S9).

References:

(1) Al-Massarani, S. M.; El-Gamal, A. A.; Al-Said, M. S.; Abdel-Kader, M. S.; Ashour, A. E.; Kumar, A.; Abdel-Mageed, W. M.; Al-Rehaily, A. J.; Ghabbour, H. A.; Fun, H.-K., *Pharmacogn. Mag.* **2016,** *12*, 114-119.

(2) Viegelmann, C.; Parker, J.; Ooi, T.; Clements, C.; Abbott, G.; Young, L.; Kennedy, J.; Dobson, D. A.; Edrada-Ebel, R., *Mar. Drugs* **2014,** *12*, 2937-2952.

(3) Panzica, R. P.; Rousseau, R. J.; Robins, R. K.; Townsend, L. B., *J. Am. Chem. Soc.* **1972,** *94*, 4708-4714.

(4) Huang, H.; Chu, C. K., *Synth. Commun.* **1990,** *20*, 1039-1046.

(5) Abe, F.; Yamauchi, T., *Phytochemistry* **1993,** *33*, 1499-1501.

(6) Hisamoto, M.; Kikuzaki, H.; Ohigashi, H.; Nakatani, N., *J. Agric. Food Chem.* **2003,** *51*, 5255-5261.
